# ADMET-XSpec: A Tool for Systematic Cross-Species Data Integration in ADMET Prediction

**DOI:** 10.1021/acs.chemrestox.5c00562

**Published:** 2026-07-20

**Authors:** Hubert Rybka, Konrad Masztalerz, Sabina Podlewska

**Affiliations:** † Faculty of Chemistry, 201871 Jagiellonian University, Gronostajowa 2, Kraków 30-387, Poland; ‡ Doctoral School of Exact and Natural Sciences, Jagiellonian University, Łojasiewicza 11, Kraków 30-348, Poland; § Maj Institute of Pharmacology, Polish Academy of Sciences, Smętna 12, Kraków 31-343, Poland; ∥ Faculty of Mathematics and Computer Science, Jagiellonian University, Prof. S. Łojasiewicza 6, Kraków 30-348, Poland

## Abstract

The rapid expansion of in silico methodologies has reshaped modern drug discovery and toxicology research; however, robust prediction of ADMET endpoints remains limited by the scarcity and heterogeneity of experimental data. In particular, toxicological data sets are often fragmented across species, complicating the development of reliable and generalizable machine learning models. To address this challenge, we introduce a dedicated Python-based computational package, ADMET-XSpec, designed for the systematic development, training, and evaluation of ML models with an explicit consideration of interspecies data integration. The framework enables controlled incorporation of chemical space originating from different species and different assay types, allowing users to flexibly construct single-species models, as well as models augmented with cross-species information. This design facilitates systematic investigation of how additional data from other organisms influence model performance without imposing assumptions inherent to specific transfer learning paradigms. By supporting standardized preprocessing, scalable integration of heterogeneous data sets, and rigorous benchmarking, the proposed tool provides a unified environment for studying cross-species effects in ADMET modeling. Overall, this work delivers a practical resource for the ADMET modeling community and offers insights into how interspecies and interassay data integration can improve model robustness and generalizability while clarifying the conditions under which cross-species and cross-assay data information is beneficial for predictive toxicology. The package is freely available at https://github.com/hubertrybka/admet-xspec. ADMET-XSpec advances the state of the art by providing the first dedicated framework for controlled interspecies and interassay data integration in ADMET modeling, offering quantitative guidance on when and how cross-species and cross-assay data improve predictive performance.

## Graphical abstract

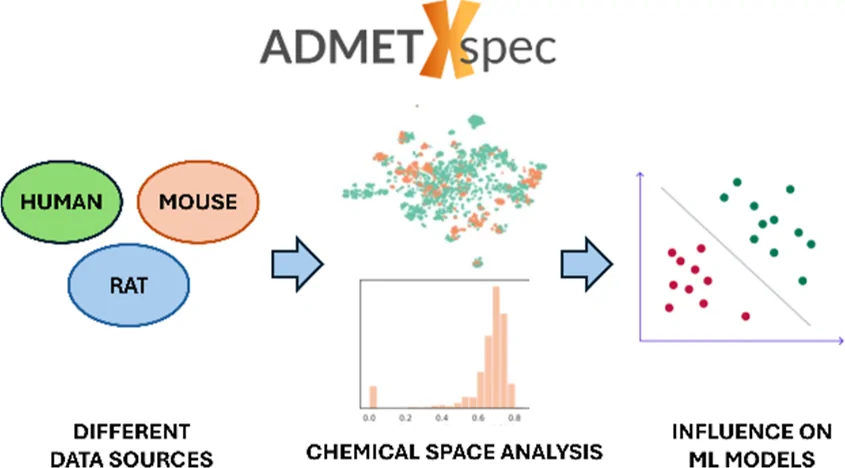

## Introduction

The exponential growth of in silico methodologies has profoundly transformed modern drug discovery. Computational tools are now embedded in nearly every stage of the pipeline, from the initial identification of potential binders to target proteins,^1–4^ through lead optimization, and finally to preclinical assessment.^5,6^ Beyond predicting binding affinity, in silico methods play a crucial role in optimizing physicochemical and pharmacokinetic properties to reduce the risk of late-stage attrition.^7–9^ Among these, the accurate prediction of absorption, distribution, metabolism, excretion, and toxicity (ADMET) properties remains one of the most critical and challenging tasks.^10,11^

However, machine learning (ML)-based tools, in order to provide reliable predictions and correctly evaluate novel compounds across vast chemical space, require training on sufficiently large and representative data sets.^12,13^ Unfortunately, in the case of ADMET properties, such data sets are often scarce or incomplete.^14,15^ Moreover, additional challenges arise from the heterogeneity of available data, as different assays are frequently conducted under varying experimental conditions, using diverse protocols and endpoints. This inconsistency hampers model robustness, limits reproducibility, and complicates the integration of results into unified predictive frameworks.^16,17^

Various strategies have been proposed to mitigate the limitations imposed by data scarcity and heterogeneity, including careful data set curation, descriptor selection, and the integration of experimental results originating from different assays or species. A systematic understanding of how such heterogeneous data can be combined effectively remains limited, particularly in the context of toxicological and pharmacokinetic modeling. Differences between species, assay protocols, and endpoint definitions may substantially influence model behavior and predictive performance, underscoring the need for controlled and transparent evaluation frameworks.

Within this broader methodological landscape, transfer learning, multitask learning, and domain adaptation represent complementary approaches to addressing insufficient data by reusing information learned from related data sets, tasks, or domains.^18,19^ For example, GeneralizedDTA pretrains its models on large unlabeled protein sequences and molecular graphs of small molecules and then fine-tunes the models on labeled binding data.^20^ Another example of binding affinity predictions enhanced by transfer learning is SubMDTA, which pretrains a graph encoder for molecules by maximizing mutual information between whole-molecule graphs and their subgraphs, then encodes proteins using a multiscale BiLSTM on n-gram amino-acid sequences.^21^ There are also approaches which transfer knowledge from chemical–chemical and protein–protein interaction tasks to drug–target affinity prediction.^22–28^ Transfer learning has also been applied to molecular property prediction in the multifidelity setting, where models pretrained on lower-fidelity data are fine-tuned on scarce high-fidelity end points.^29^ Also, the graph transformer-based architectures have been shown to benefit from transfer learning for the prediction of physicochemical properties such as aqueous p*K*_a_.^30^ There are also a number of examples where transfer learning has been used for ADMET prediction tasks.^28,31^ Although transfer learning in its current, formalized sense dates back only a decade or so, the underlying idea is considerably older. Conceptually, transfer-learning-like strategies have been explored for over two decades, for example, in an early work applying associative neural networks pretrained on public lipophilicity data to correct predictions using proprietary in-house log *P*/log *D* measurements.^32,33^

Multitask learning offers a related but distinct strategy, training a single model simultaneously on multiple related end points to exploit shared structural and biological information across tasks.^34,35^ For example, Ramsundar et al. proposed massively multitask neural networks, showing that training on large collections of biological assays (∼40 M measurements across >200 targets) improves predictive performance over single-task models.^36^ It was then further indicated that the incorporation of additional information (such as evolutionary distance between targets) can improve the prediction power of the models.^37^ The applicability of multitask learning has been demonstrated across diverse settings, including multiorgan toxicity modeling^38^ and natural product bioactivity prediction under severe data limitations.^39^

Domain adaptation is another strategy in this field that addresses data scarcity. This approach explicitly accounts for distributional shifts between source and target data sets and has already demonstrated successful applications in compound property prediction.^40,41^

Despite the above-described advances, the applicability and practical benefits of transfer and multitask learning in ADMET modeling, particularly in the presence of interspecies variability, remain incompletely understood. Differences in biological systems, assay protocols, and end point definitions may substantially influence model behavior, and it is often unclear whether performance gains arise from meaningful knowledge transfer or from implicit biases introduced by heterogeneous data sources. This ambiguity highlights the need for systematic frameworks that enable the controlled investigation of how data originating from different species can be incorporated into predictive models and how such integration affects model performance.

To address these challenges, we introduce an ADMET-XSpec, dedicated computational package designed for systematic development, training, and evaluation of ML models with explicit consideration of cross-species data integration. This Python-based framework enables flexible construction of single-species models as well as models augmented with data from other species, allowing for controlled analysis of interspecies effects. The following properties were considered in this study: compound metabolic stability expressed as half-life (*t*_1/2_), monoamine oxidase A (MAO-A) affinity, and acetylcholinesterase (AChE) inhibition. To further assess the applicability of the framework beyond interspecies integration, blood–brain barrier (BBB) permeability was additionally included as a case study in which data originating from two experimentally distinct assay types were combined within the same modeling pipeline. These properties capture key determinants of a compound’s pharmacokinetic and pharmacodynamic behavior. Metabolic stability influences how long a molecule can remain active in the body, while blood–brain barrier permeability determines whether it can effectively reach the central nervous system.^42,43^ MAO-A affinity provides insight into potential off–target interactions affecting neurotransmitter metabolism,^44,45^ and AChE inhibition reflects a compound’s ability to modulate cholinergic signaling. Because AChE inhibition is also a well-established marker of neurotoxic risk, due to its direct role in acetylcholine breakdown and potential to disrupt neural communication, it serves as an important endpoint for evaluating both therapeutic relevance and safety.^46,47^

ADMET-XSpec enables the controlled incorporation of data originating from different species based on the degree of chemical similarity to a reference data set, allowing for flexible construction of single-species models as well as models augmented with interspecies or interassay data. Users can precisely select chemical representations, control the extent and composition of the chemical space included, train ML models, and visualize the resulting distribution of data. This functionality facilitates the systematic and transparent exploration of how cross-species data integration influences model behavior and predictive performance in ADMET modeling. The package is freely available at https://github.com/hubertrybka/admet-xspec.

## Materials and Methods

### Data Set Preparation

All data used in the study were fetched from the ChEMBL database,^48^ apart from the BBB and PAMPA (Parallel artificial membrane permeability assay) permeability data, which were sourced independently. BBB permeability data was fetched from the B3DB database, and PAMPA permeability data was collected from Harvard dataverse.^49,50^ Assays referring to the considered properties (stability (*t*_1/2_), MAO-A inhibition, and AChE inhibition) were searched for, with reference to three species: human (*Homo sapiens*), rat (*Rattus norvegicus*), and mouse (*Mus musculus*). The specific ChEMBL assay IDs considered in the study are summarized in Supporting Information (ZIP File). The data gathered from ChEMBL was filtered to retain only data points with the following parameters: Standard Type = “IC50”, Standard Unit = “nM” for MAO-A and AchE data sets and Standard Type = “*T*_1/2_”, Standard Unit = “hr” for hepatic stability data set. For the training of regression models, only data with exact labels (Standard Relation = “=”) were used. All the molecules were stripped of counterions and had their charges neutralized as a preprocessing step. Continuous target values were transformed by applying a negative logarithm before training.

### ML Models

Our tool enables systematic exploration of factors influencing knowledge transfer between different species. Initially, compounds were represented using multiple molecular representations, including extended-connectivity fingerprints (ECFP4),^51^ molecular ACCes system (MACCS)^52^ fingerprints, and a set of physicochemical descriptors calculated with RDKit version 2025.3.6 (detailed data are provided in the GitHub repository: https://github.com/admet-xspec/admet-xspec/blob/main/src/data/featurizer.py).^53^ The data set was divided into training and test sets in a 0.8:0.2 ratio using two splitting strategies: random splitting and scaffold-based splitting (the exact sizes of each data set are provided in the Supporting Information (File S1)). In the scaffold-based approach, all compounds were first grouped according to their Bemis-Murcko scaffold.^54^ Next, from all the resulting groups, a random group of molecules is selected and appended to the training set. If the size of the resulting training set is larger than 80% of the original data set, the group is appended to the test set instead. This continues until all of the scaffold groups are assigned. The test set always consisted exclusively of human data and was used to evaluate how the inclusion of data from other species in the training set affected prediction performance. Compounds from nonhuman species were incorporated into the training set in a stepwise manner, separately for mouse, rat, and a combined mouse + rat scenario.

Inclusion of nonhuman compounds was controlled by a similarity criterion: a nonhuman compound is included in the augmented training set if its Tanimoto similarity to the most similar compound in the human data set is below or equal to the predefined threshold. This threshold was treated as a user-defined parameter, allowing for control over the degree of structural overlap between the data added from different species.

ML models employed in this study included random forest (RF),^55^ support vector machines (SVM) with RBF kernel,^56^ and lightGBM.^57^ The hyperparameters of the models were tuned on data sets containing human data exclusively. The hyperparameters of the models were tuned according to the following strategy: a tree-structured Parzen estimator method, implemented in Optuna library, was used to perform 100 iterations of hyperparameter sampling from a predefined distribution. The performance of the resulting models was evaluated using a 5-fold cross-validation strategy with ROC AUC as the target metric for classifier models and MSE as the target metric for regressors. The hyperparameter distributions used in this study, together with versions of all packages used and split statistics, are presented in the Supporting Information (File S1). Confidence intervals for all metrics were evaluated with the bootstrap method, resampling the test set 1000 times, and are given as a 95% confidence interval. The total number of ML models prepared for this study was 5517.

Experiments were carried out in two modes: classification and regression. For classification tasks, the data samples obtained from the ChEMBL database were assigned binary classes based on their relation to the following threshold values; 1 μM for AChE and MAO-A inhibition and 0.5 h for liver *t*_1/2_.

## Results and Discussion

### Data Analysis

The data distribution was analyzed by using several complementary approaches. First, the distribution of individual parameter values was examined (Figure 1). The presented distributions clearly indicate a high degree of similarity across different species (human, rat, and mouse). For all considered properties, the peaks of the distributions occur at comparable parameter values. The widths of the distributions are also largely similar across species, particularly *t*_1/2_ and AChE-related data sets. In the case of MAO-A, the distribution corresponding to rat data is noticeably broader and flatter compared with the human data set, although the peak occurs for a similar value of IC_50_. For plasma permeability, the data sets were constructed differently. The B3DB data set was complemented with experimental PAMPA data. The class distribution differed between these data sets: the B3DB data set contained a substantially higher proportion of positive (permeable) examples than the PAMPA data set. While B3DB reflects an aggregated BBB permeability assessment based on diverse experimental sources, PAMPA constitutes a straightforward artificial membrane assay routinely applied in early-stage screening. In practice, compounds failing PAMPA permeability criteria are usually excluded from subsequent, more advanced BBB evaluation assays, which may explain the lower fraction of permeable compounds observed in the B3DB data set. The BBB data set was included in the study to demonstrate that the framework presented within the ADMET-XSpec extends beyond the examination of cross-species integration and can systematically assess the impact of combining any heterogeneous data sources on the predictive model performance.

**Figure 1:**
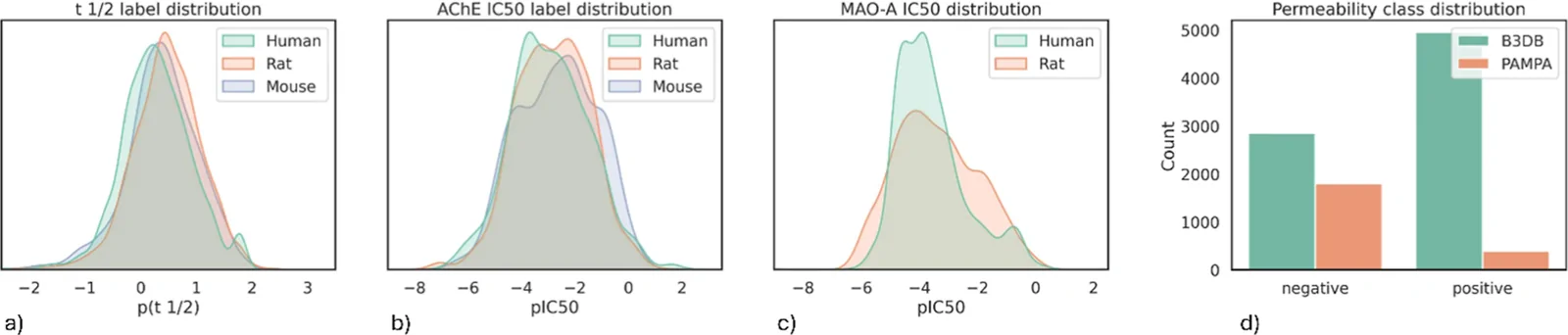
Distribution for human, rat, and mouse data for compound parameters considered in the study (a) *t*_1/2_, (b) AChE inhibition, (c) MAO-A inhibition, and (d) plasma permeability.

In addition to the analysis of individual parameter distributions, the coverage of chemical space by the compounds in each considered data set was assessed via t-SNE analysis; for this analysis, classification data on ECFP4 fingerprints were taken into account (Figure 2).

**Figure 2:**
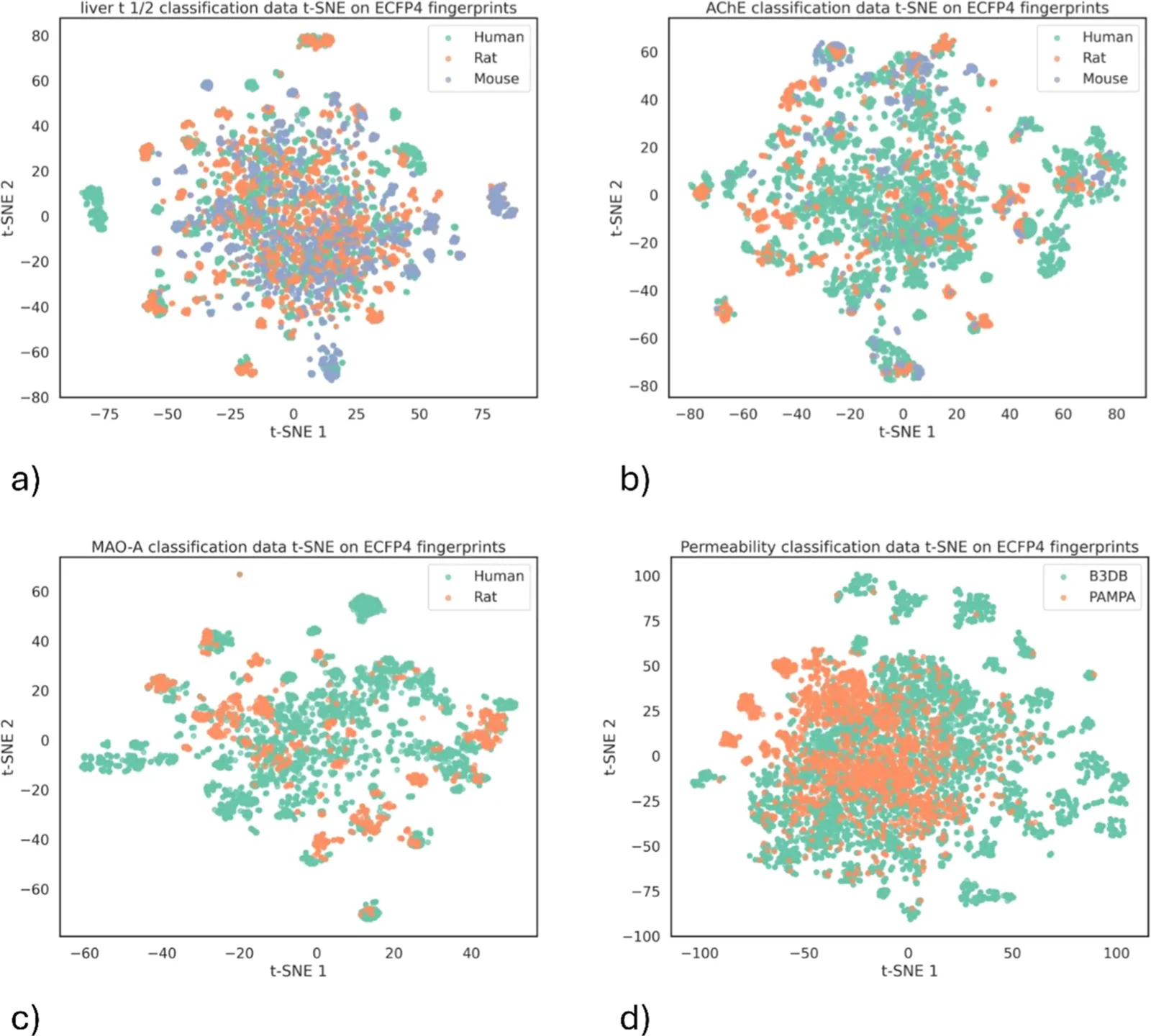
Distribution of compound structures for human, rat, and mouse used in the study for (a) *t*_1/2_, (b) AChE inhibition, (c) MAO-A inhibition, and (d) BBB permeability.

The results indicate that although compounds tested in assays based on different species generally occupy a similar chemical space, several distinct clusters can be identified. These clusters represent ligands exploring different regions of structural space and may be particularly important for the transferability of results obtained from different species in ML-based prediction models.

In the *t*_1/2_ data, several data points are located in distinct regions of the t-SNE space. For example, two separate clusters of mouse data are visible and located quite far from the chemical space occupied by the remaining data. In addition, one distinct cluster of human data can be observed that is not close to any other clusters. Several other smaller regions can also be distinguished in the t-SNE plot. In contrast, AChE data show a much higher degree of structural overlap between species. No compounds specific to a single species appear to be structurally distinct from all other data, indicating a shared chemical space across species. For MAO-A, only human and rat data were available in sufficient quantities for the analysis. When these data sets are compared, regions dominated by human compounds (green points) can be observed where no nearby rat compounds (orange points) are present. However, rat data points are generally located close to human data points despite the presence of three more clearly separated groups within the overall distribution. In the t-SNE analysis of permeability data, the situation differs due to the nature of the data set. Distinct regions occupied predominantly by either human (green) or rat (orange) compounds were observed. As mentioned earlier, compounds with negative PAMPA results were most likely not subjected to further BBB assays, which likely contribute to this separation.

### Tanimoto-Based Analysis of the Augmented Data

As this study is based on data set augmentation through the incorporation of compounds from other species according to their Tanimoto similarity to the training data, the data sets were analyzed with respect to the chemical similarity between compounds originating from different augmented subsets and the original training set (Figure 3). This analysis enabled a quantitative assessment of how closely the additional interspecies data resembled the reference chemical space used for the model development. The analysis was performed separately for classification and regression data sets, as differences in data set construction resulted in slight variations between these two data types.

**Figure 3:**
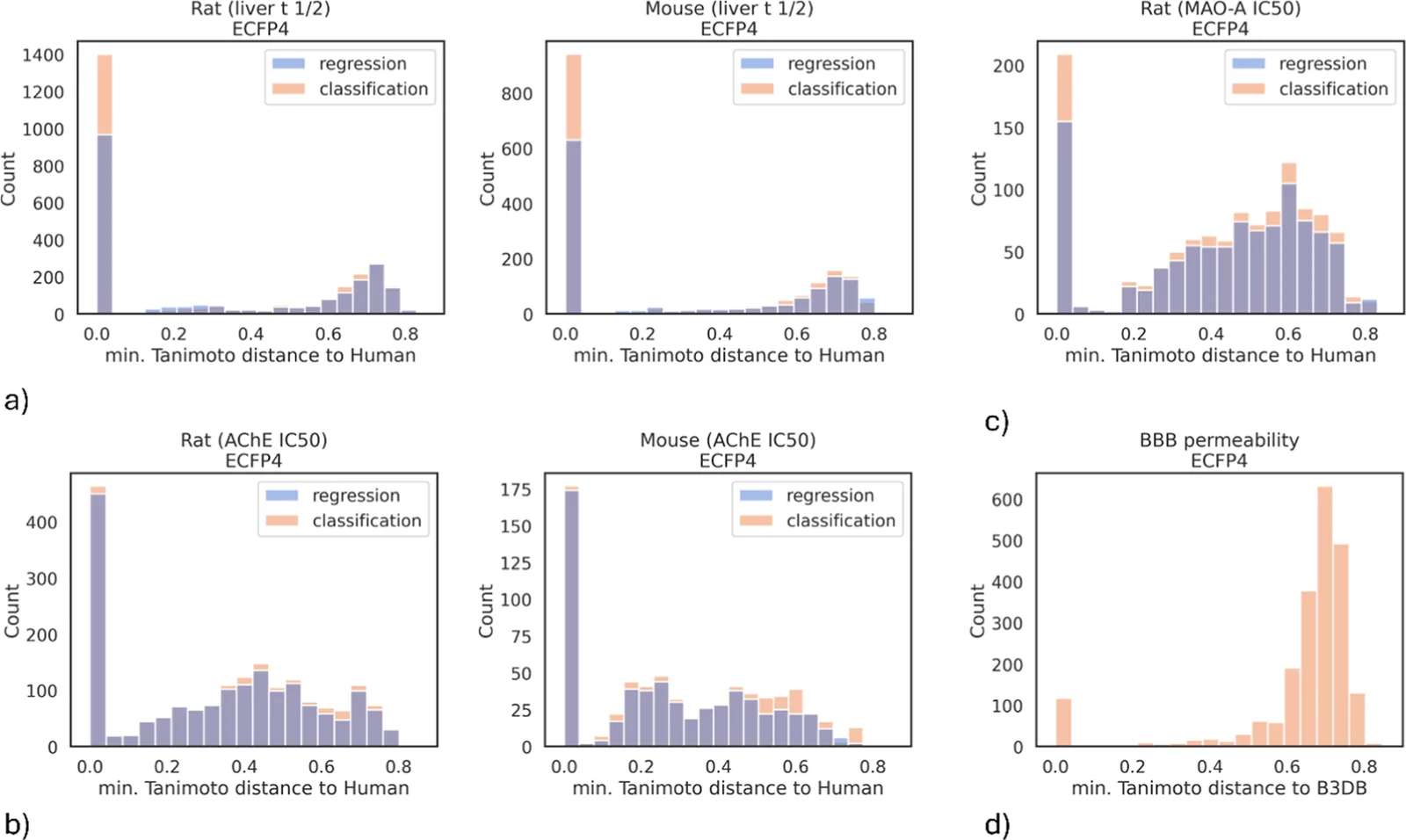
Distribution of Tanimoto distances to the human training data for rat and mouse data for (a) *t*_1/2_, (b) AChE inhibition, (c) MAO-A inhibition, and (d) BBB permeability.

Across all studied endpoints, a substantial number of compounds exhibited exact overlap between augmented subsets and the original training data, corresponding to a Tanimoto distance of 0. The number of such overlapping compounds ranged from more than 150 in the mouse AChE data set to approximately 1400 in the rat *t*_1/2_ data set. Notably, the similarity distributions were more strongly influenced by the considered ADMET endpoint than by the species from which the data originated. For a given property, the overall shape of the Tanimoto similarity distribution was largely consistent across the species. For example, in the case of *t*_1/2_ data, only a small number of compounds exhibited intermediate similarity values (excluding exact matches) within the Tanimoto distance range of 0 to approximately 0.5. The number of compounds then increased gradually, reaching a maximum at a similarity of around 0.75 for both rat and mouse data sets.

In contrast, AChE inhibition data did not display a clear unimodal or Gaussian-like similarity distribution. Instead, the number of compounds identified at specific Tanimoto similarity thresholds varied irregularly, ranging from approximately 50 to 150 for rat data and from about 25 to 50 for mouse data without a discernible trend across similarity values.

For MAO-A affinity, the highest concentration of compounds was observed at a Tanimoto similarity of approximately 0.6, whereas for blood–brain barrier permeability data, the maximum peak occurred at a similarity of around 0.7.

Overall, the t-SNE projections reveal discrete clusters corresponding to nonhuman compounds, confirming that the high Tanimoto distance tail observed in the histogram analysis reflects genuine expansion of chemical space beyond that covered by the human data set alone.

### Interspecies Consistency of In Vitro Data

The consistency of assay outcomes across species was further examined. To this end, Venn diagrams were constructed to quantify the overlap of compounds tested in assays conducted for different species (Figure 4). Here, we focus on the analysis of human liver metabolic stability expressed as half-life (*t*_1/2_); the complete set of results is provided in the Supporting Information.

**Figure 4:**
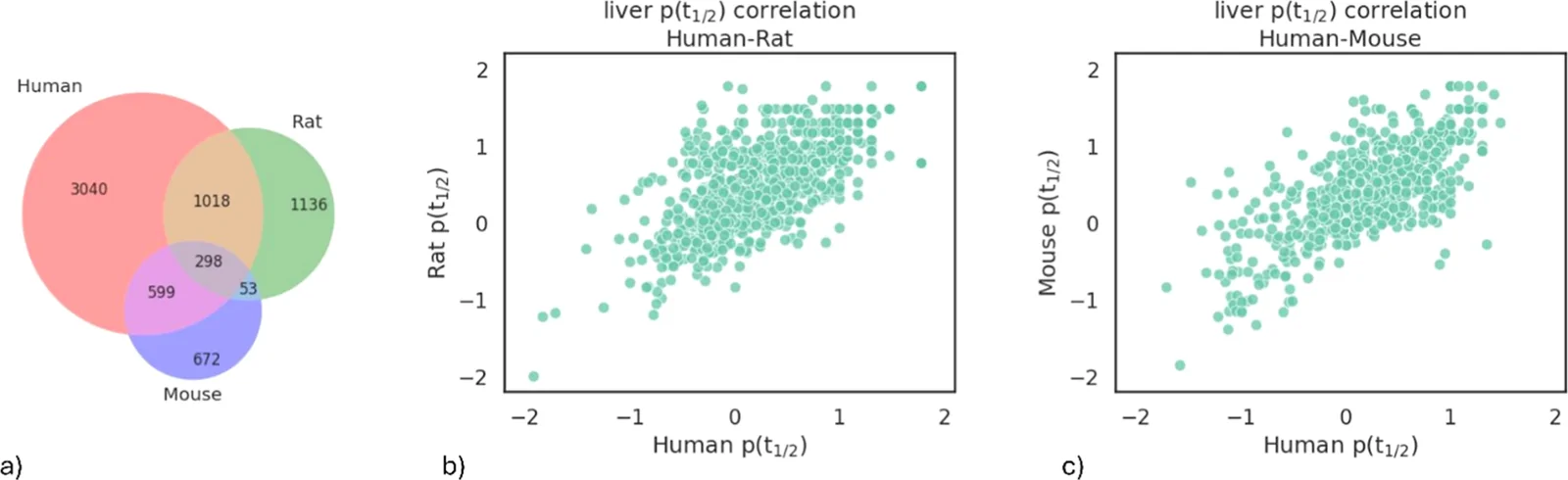
Interspecies consistency for the *t*_1/2_ data. (a) Venn diagram (b) human-rat data correlation, and (c) human-mouse data correlation.

For the *t*_1/2_ data set, a substantial fraction of compounds (ranging from approximately 25% up to nearly 60%) was evaluated in assays performed for more than one species. Notably, almost 300 compounds were tested across all three species (human, rat, and mouse). Despite this considerable overlap, the correlations between human-rat and human-mouse stability data were far from ideal. The corresponding correlation plots reveal a broad dispersion of data points around the line of perfect correlation, indicating a pronounced interspecies variability. This observation reflects a relatively large number of compounds for which in vitro metabolic stability measurements differ substantially when transitioning from human to rat or mouse data sets.

### ML Models-Tanimoto-Dependent Relationships

The results presented here are based on the scaffold-split scenario, which most closely approximates external validation by ensuring evaluation on structurally novel chemical space; confidence intervals estimated via bootstrap (1000 iterations) are reported for all data points. A Tanimoto-based filter threshold of 0.0 corresponds to inclusion of cross-species data for structurally identical compounds only. Classification and regression performance are reported as ROC-AUC and MSE, respectively. Each figure presented in the results section shows three conditions: human-only baseline (dashed blue), naïve augmentation without source encoding (red), and attributed augmentation with explicit species identity encoding (green).

This section focuses on the general qualitative trends observed across benchmark analyses. A detailed discussion of selected representative examples, as well as the complete quantitative results, is provided in the Supporting Information (DOCX File, ZIP File).

### *T*_1/2_ Liver (LightGBM)

The impact of cross-species data integration for LightGBM was strongly endpoint-, representation-, and threshold-dependent, and augmentation was not universally beneficial (Figures 5–7). A consistent finding across all representations for LightGBM was that compounds with Tanimoto distance ≥0.6 to the human training set contributed negligibly to model performance, which can be considered, therefore, as a practical upper bound for productive cross-species data incorporation for these scenarios. At *T*_c_ distance = 0.0 (where structurally identical cross-species compounds are present), the choice of augmentation strategy was critical. The effect of naïve augmentation on models’ predictive power was not uniform across representations and species combinations. For ECFP-based and RDKit Descriptors-based models, naïve augmentation with rat data increased MSE by approximately 0.04, whereas for MACCSFP, the MSE was reduced by ∼0.03 upon analogous augmentation. When additional mouse data were incorporated (rat + mouse scenario), ECFP-based models showed MSE values comparable to those obtained using human data only. In contrast, MACCS fingerprints resulted in a decrease in MSE of approximately 0.04, whereas RDKit descriptors exhibited an increase of ∼0.02. Similar trends were observed for classification models, with naïve augmentation with rat (and subsequently rat + mouse) data, leading to AUC-ROC drops of approximately 0.1 and 0.2 for ECFP- and RDKit descriptors-based models, respectively, and an ROC-AUC improvement of ∼0.01 for MACCS fingerprint-based models.

**Figure 5:**
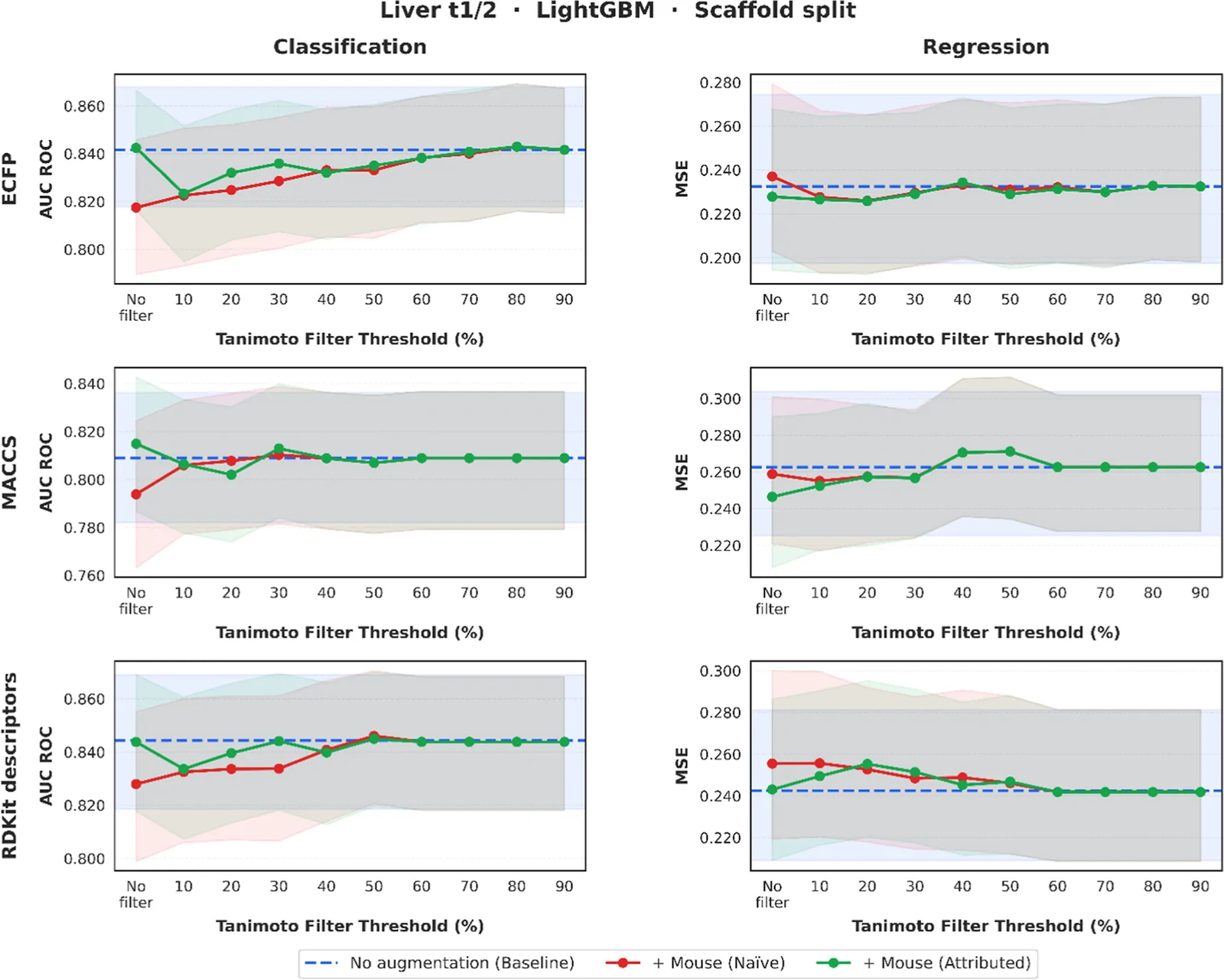
Analysis of the influence of human-data-based training set augmentation with mouse data on the LightGBM-based model performance predicting *t*_1/2_ liver. Shaded areas represent 95% bootstrap confidence intervals.

**6:**
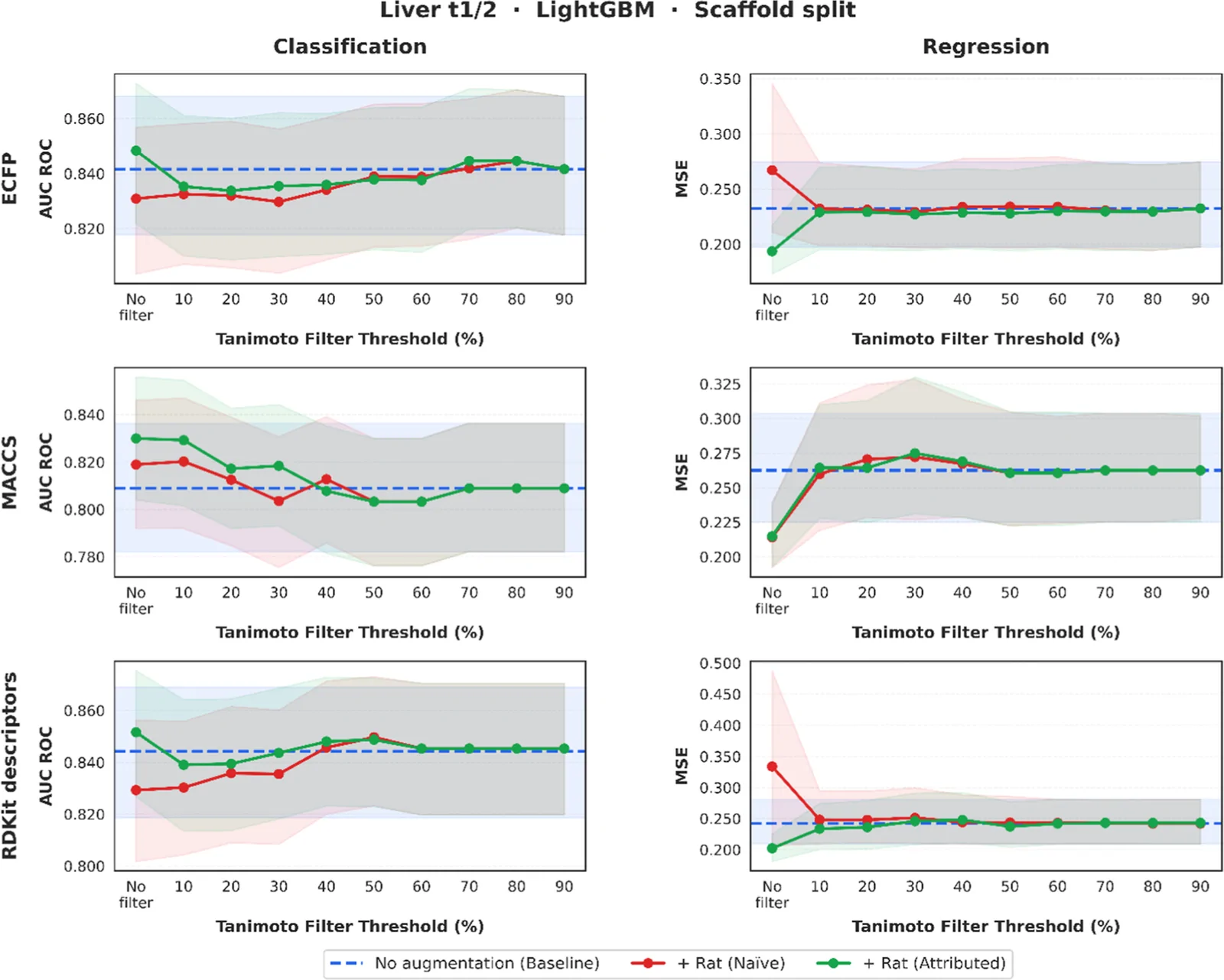
Analysis of the influence of human-data-based training set augmentation with rat data on the LightGBM-based model performance predicting *t*_1/2_ liver. Shaded areas represent 95% bootstrap confidence intervals.

**Figure 7:**
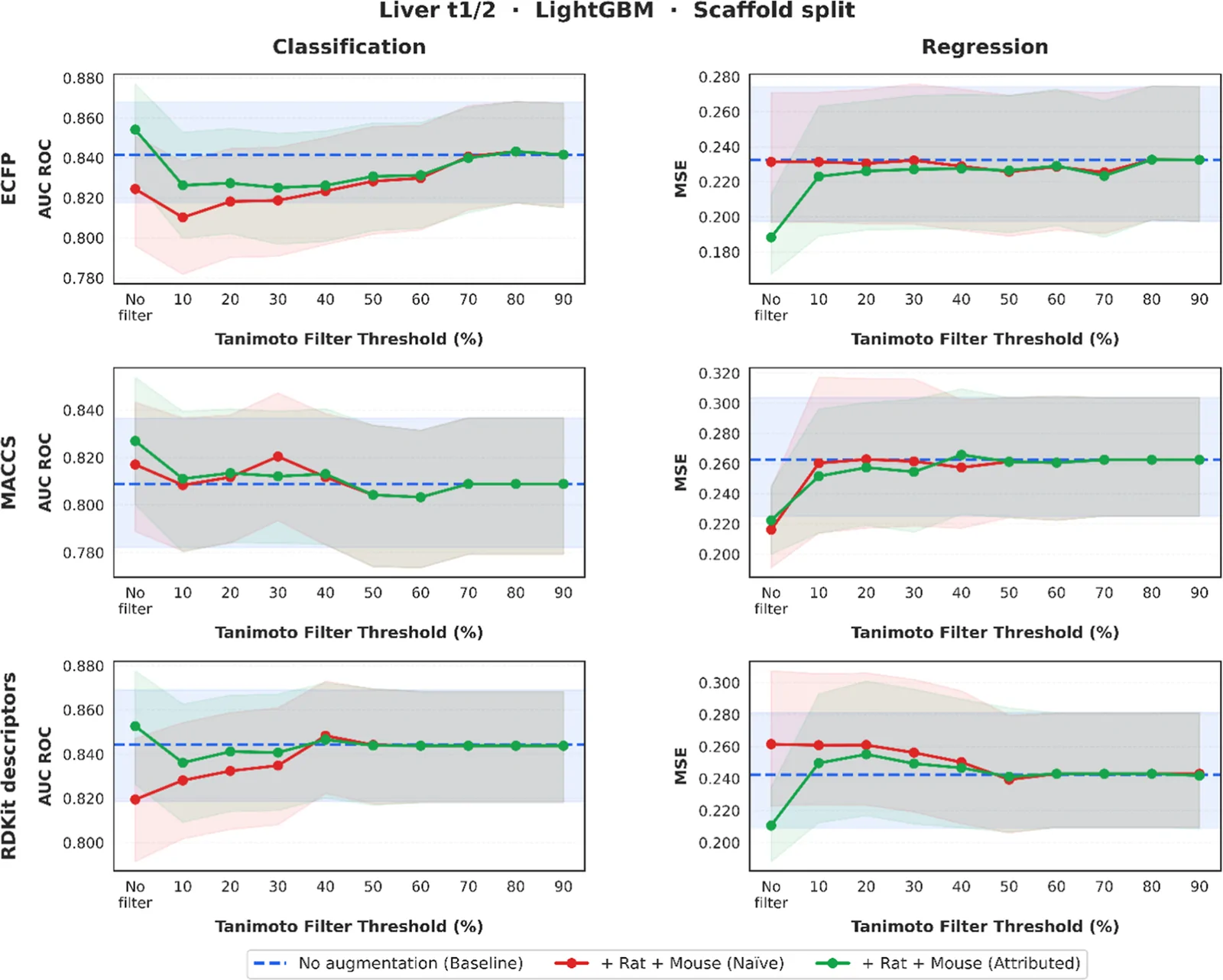
Analysis of the influence of human-data-based training set augmentation with rat and mouse data on the LightGBM-based model performance predicting *t*_1/2_ liver. Shaded areas represent 95% bootstrap confidence intervals.

Attributed augmentation for lower Tanimoto thresholds (*T*_c_ = 0.1–0.4) yielded modest MSE reductions (∼0.01–0.02) for the addition of mouse data, but simultaneously a slight drop in ROC-AUC, most pronounced at *T*_c_ = 0.1 (∼0.02 for ECFP and RDKit descriptors). For the augmentation of rat data, ROC-AUC was substantially higher for MACCS fingerprints (by up to ∼0.02) but marginally lower for other representations (by 0.005–0.01) in classification experiments. When regression is considered, MSE was also higher for MACCS fingerprints in this scenario (up to 0.01 at *T*_c_ = 0.3), while for ECFP and RDKit descriptors, the influence on model performance was negligible (MSE change ≤0.002). For combined rat + mouse augmentation, attributed augmentation reduced ROC-AUC by up to 0.01–0.02, with generally larger effects for ECFP than RDKit descriptors and slightly smaller effects for MACCS fingerprints (∼0.005). MSE reductions were also observed: ∼0.015 for MACCS fingerprints and ECFP, whereas RDKit descriptor-based models showed increased MSE (∼0.1–0.15).

Overall, the Tanimoto similarity of incorporated compounds was a stronger determinant of model performance than the augmentation strategy employed, underscoring the primacy of chemical-space considerations in cross-species data integration in this particular scenario.

### *T*_1/2_ Liver (SVM and RF)

For both SVM and RF models, attributed augmentation consistently outperformed naïve data merging at *T*_c_ = 0.0 (Figures 8, 9, Supporting Information, ZIP File); for example, ECFP-based classification models yielded AUC-ROC equal to ∼0.840 (rat + mouse attributed augmentation) vs ∼0.830 (baseline); whereas in regression, ECFP-based predictions led to the following MSE values: ∼0.175 (rat + mouse attributed augmentation), ∼0.205 (naïve augmentation), and ∼0.220 (human-only baseline).

**Figure 8:**
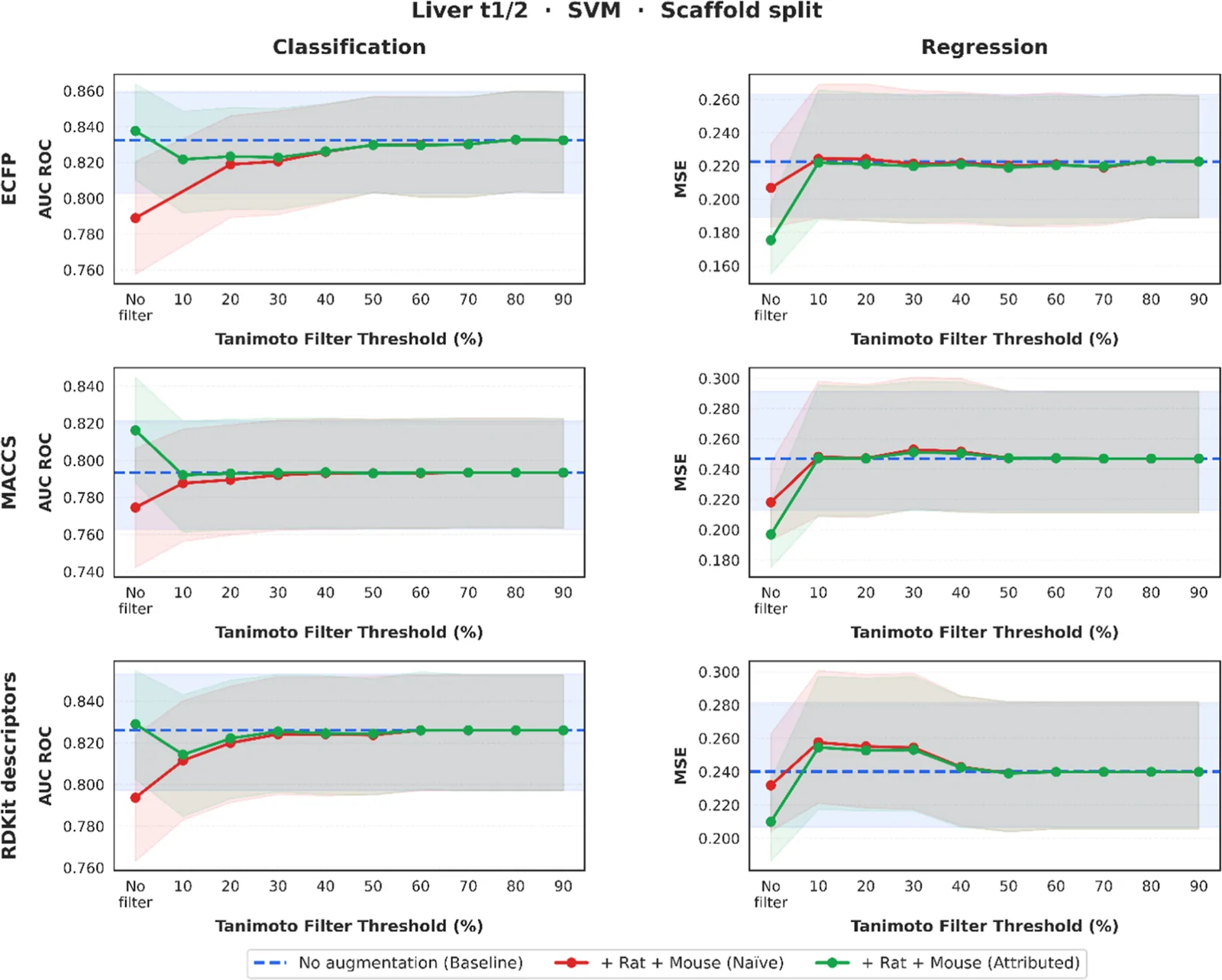
Analysis of the influence of human-data-based training set augmentation with rat and mouse data on the SVM-based model performance predicting *t*_1/2_ liver. Shaded areas represent 95% bootstrap confidence intervals.

**Figure 9:**
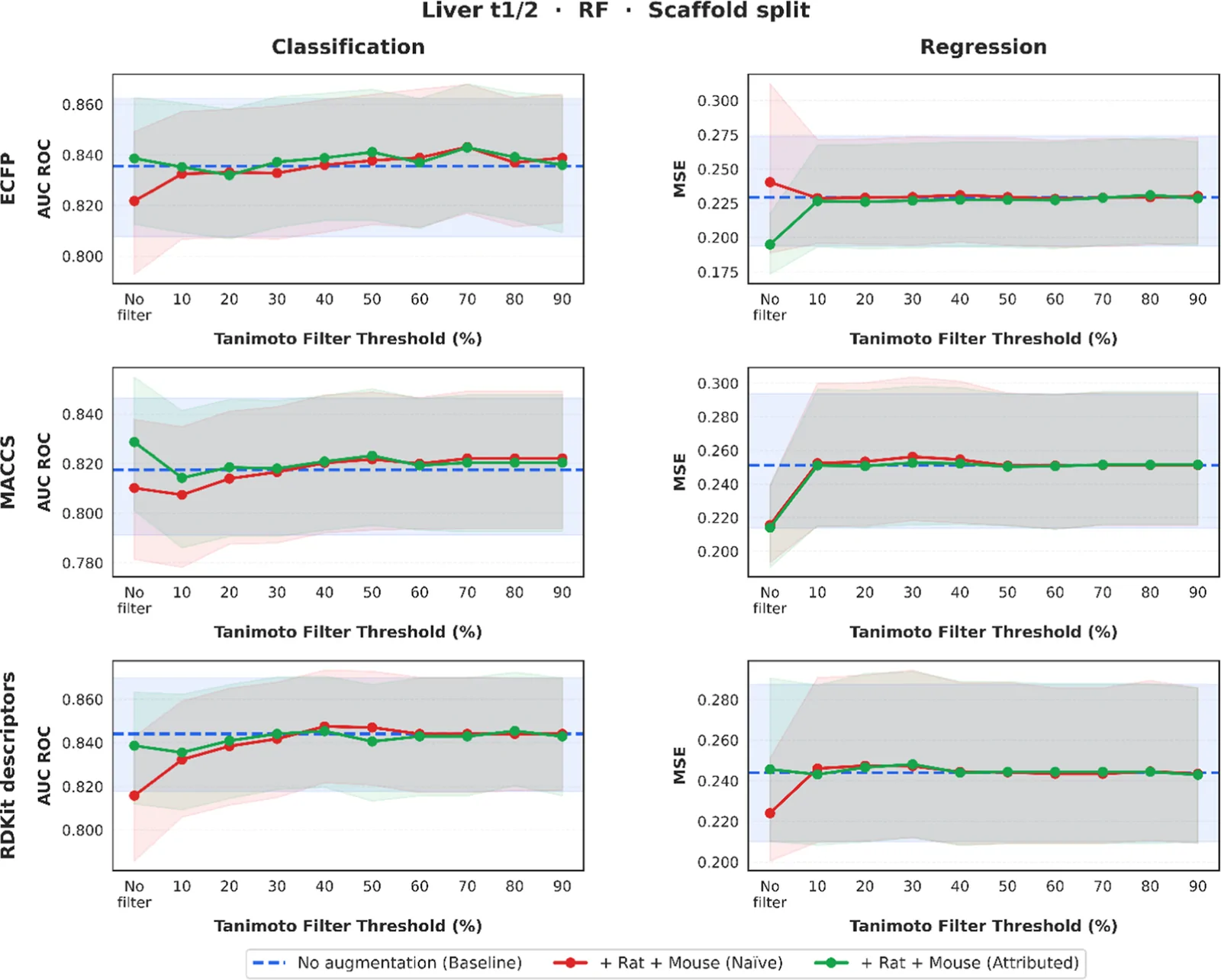
Analysis of the influence of human-data-based training set augmentation with rat and mouse data on the RF-based model performance predicting *t*_1/2_ liver. Shaded areas represent 95% bootstrap confidence intervals.

Above *T*_c_ = 0.5, augmentation had no measurable impact on SVM performance for both regression and classification tasks. In contrast, RF regression models converged to baseline performance after rat + mouse data augmentation already at *T*_c_ ≥ 0.1, while RF classification models exhibited the same behavior at *T*_c_ ≥ 0.3 for both augmentation strategies.

Mouse-only augmentation showed a distinct pattern: ROC-AUC was reduced across all strategies for *T*_c_ ≤ 0.3, with only marginal improvements (<0.005) observed for *T*_c_ > 0.5. Regression performance was similarly unaffected, with the exception of *T*_c_ = 0.0 where a negligible change in MSE (<0.005) was noted.

### AChE Inhibition

For AChE inhibition predicted by LightGBM, molecular representation was the primary driver of augmentation effects, with the choice of augmentation strategy playing a secondary role (Supporting Information, ZIP File). ECFP-based models benefited most consistently: classification ROC-AUC improved by ∼0.02 at *T*_c_ = 0.0, declining to ∼0.01 for *T*_c_ = 0.1–0.4, and converging to baseline above *T*_c_ = 0.5 and regression MSE was reduced by up to ∼0.1 for *T*_c_ ≤ 0.6. MACCS and RDKit descriptor-based models showed more variable and threshold-sensitive responses, with improvements generally confined to *T*_c_ = 0.0 and negligible or negative effects at higher thresholds.

RF and SVM models demonstrated markedly greater stability for AChE inhibition prediction across most conditions, with ROC-AUC changes below 0.01 and MSE differences below 0.02, with the exception of *T*_c_ = 0.0, where ROC-AUC changes of up to 0.02 and MSE changes of up to 0.1 were observed for SVM and ROC-AUC improvement by 0.05 and MSE reduction by 0.07 for RF (augmentation with rat and mouse data).

### MAO-A Inhibition

For MAO-A, cross-species augmentation at *T*_c_ = 0.0 was uniformly beneficial across most algorithms, representations, and species combinations, with the exception of naïve augmentation for the RF/RDKit Descriptors scenario, where no measurable improvement relative to baseline was observed (Supporting Information, ZIP File), and LightGBM showing the greatest gains. ROC-AUC improvements in classification studies ranged from ∼0.015 (MACCS fingerprints) to ∼0.03 (ECFP, RDKit descriptors), whereas MSE reductions in regression reached up to 0.15 for ECFP and RDKit descriptors. Above *T*_c_ = 0.0, the threshold above which augmentation ceased to provide measurable benefit was algorithm- and representation-dependent, ranging broadly across the tested Tanimoto range.

### BBB Permeability

BBB permeability represents a distinct case study in this work, designed to assess the framework’s applicability beyond interspecies integration by examining the effect of cross-assay data incorporation. In the presented case, we examined the addition of PAMPA-derived permeability measurements to a widely used benchmark in BBB predictions. In contrast to the endpoints discussed above, PAMPA data augmentation consistently degraded classification performance across all algorithms and representations for random splitting-based experiments and the majority of scenarios for scaffold-based splitting (Figures 10–12; Supporting Information, ZIP File). Naïve augmentation was essentially never beneficial, while attributed augmentation showed a more nuanced pattern, with AUC-ROC degradation the smallest for LightGBM (when different ML algorithms are considered) and the largest for *T*_c_ = 0.0 (when similarity of augmented data is taken into account). This outcome indicates that, in contrast to cross-species data, the two BBB assay types are too incompatible in terms of their theoretical and methodological foundations to effectively integrate them under examined conditions.

**Figure 10:**
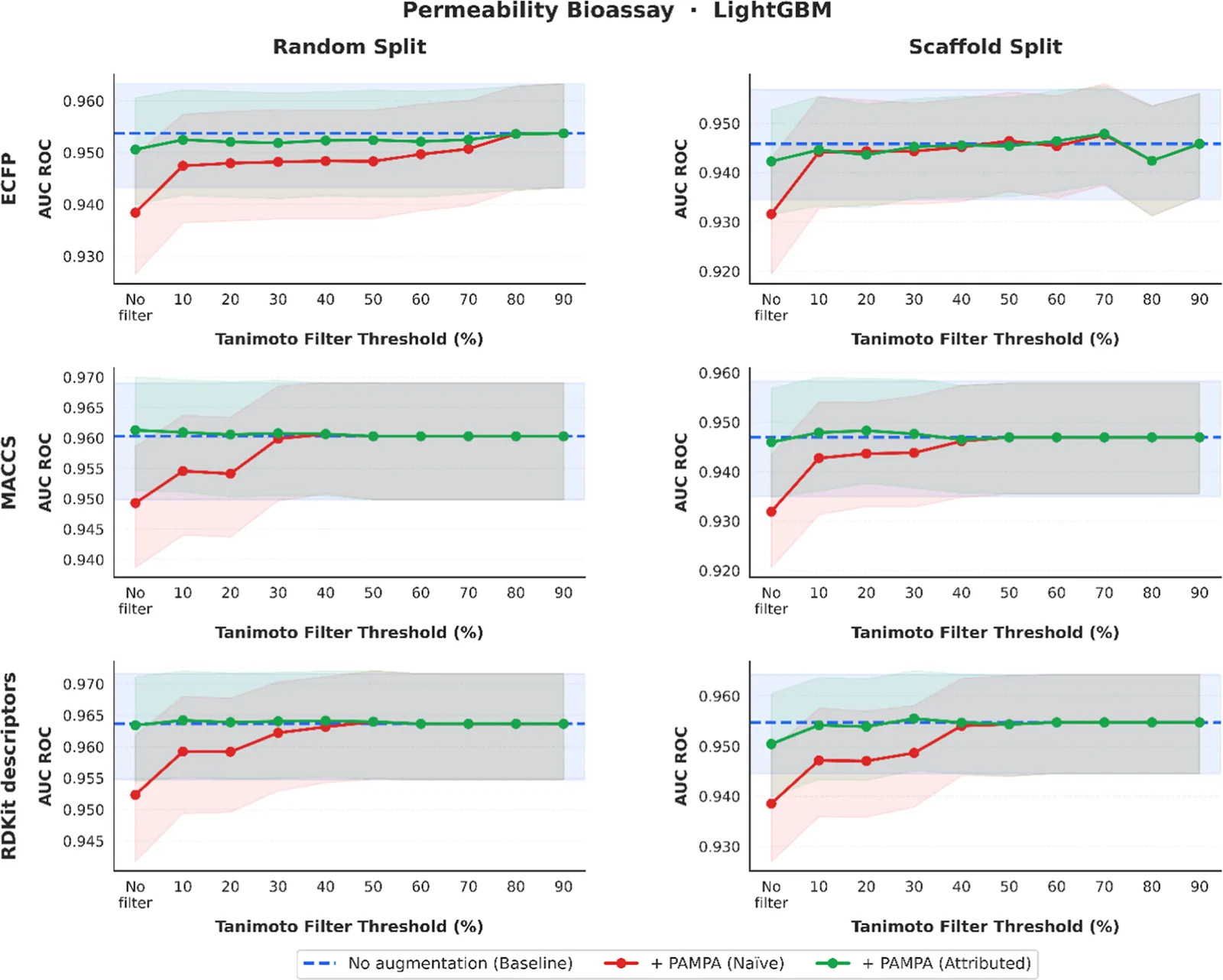
Analysis of the influence of cross-assay data integration for BBB predictions for LightGBM. Shaded areas represent 95% bootstrap confidence intervals.

**11:**
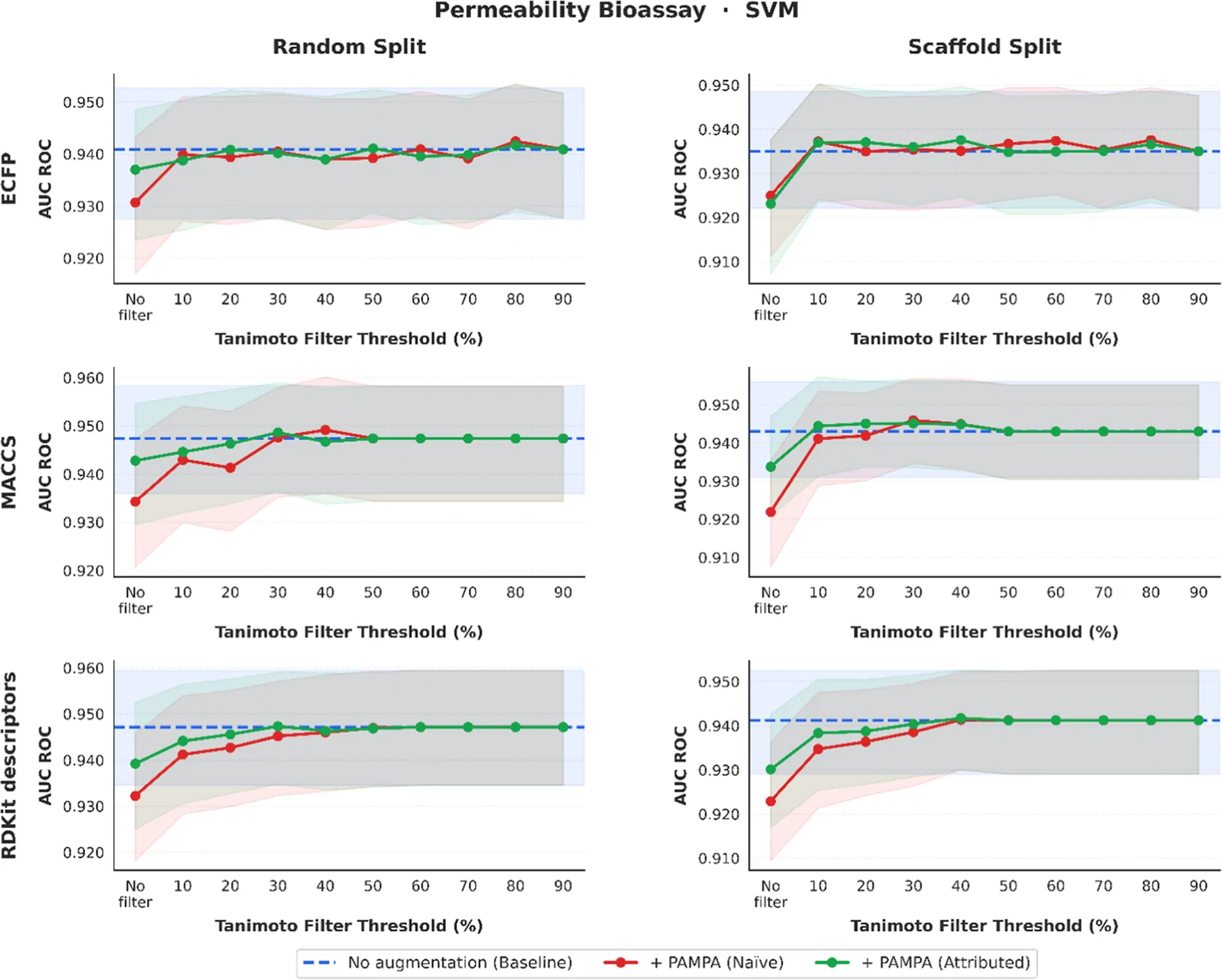
Analysis of the influence of cross-assay data integration for BBB predictions for SVM. Shaded areas represent 95% bootstrap confidence intervals.

**Figure 12:**
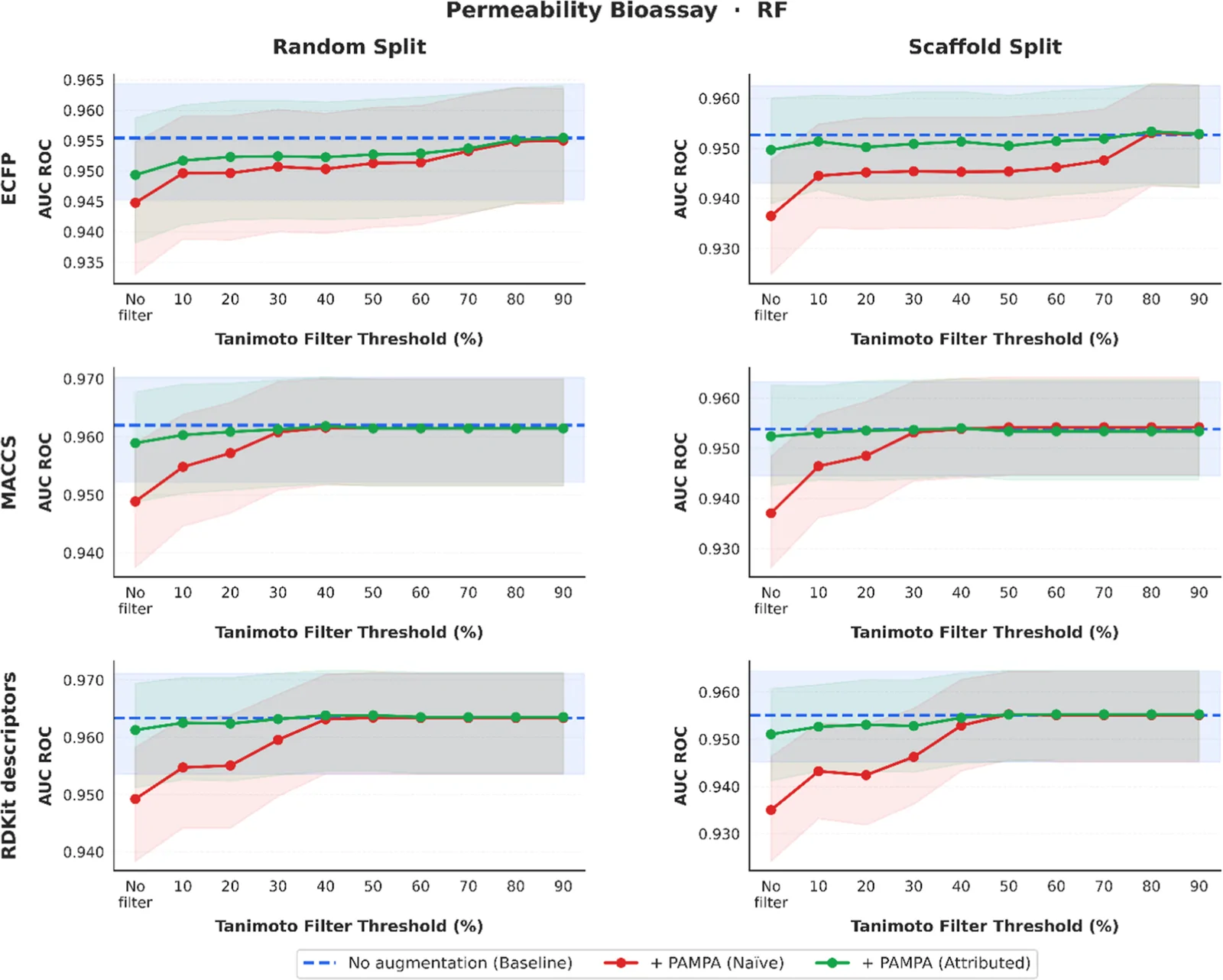
Analysis of the influence of cross-assay data integration for BBB predictions for RF. Shaded areas represent 95% bootstrap confidence intervals.

### General Comments

The results presented in this study demonstrate that the impact of cross-species and cross-assay data integration on ADMET model performance is strongly context-dependent, varying across endpoints, molecular representations, ML algorithms, and Tanimoto similarity thresholds. No universal augmentation strategy consistently outperformed the human-only baseline across all evaluated conditions, underscoring the futility of prescriptive, one-size-fits-all approaches to heterogeneous data integration.

As no universal augmentation strategy emerged from the obtained results, the cross-species augmentation should be treated as an empirical option which needs to be tested for each condition rather than being a default choice. In addition, explicit source-domain encoding (attributed augmentation) should be preferred over naïve data merging whenever structurally identical or near-identical compounds are present across species (*T*_c_ = 0.0) since this is precisely the scenario in which conflicting labels are most likely and where attributed augmentation is in most cases preferential over other strategies. Finally, above *T*_c_ ∼ 0.6, the cross-species compounds contribute negligibly to model performance, which can be a practical upper bound for data integration strategies.

The BBB permeability case study further illustrates the limits of cross-assay integration but also the broad applicability of the ADMETXSpec framework. Incorporation of PAMPA data consistently degraded benchmark-based permeability model performance across all conditions, likely reflecting the fundamental physicochemical differences between transport mechanisms operative in biological BBB models. This result highlights that assay compatibility must be carefully considered when designing the augmentation strategies.

While attributed augmentation generally outperformed naïve data merging, the absolute magnitude of improvement was, in many cases, modest (typically on the order of 0.01–0.05 in ROC-AUC or MSE). Whether such gains are practically meaningful depends on the intended use of the model. For early stage compound triage or virtual screening, where models are used to rank large libraries and only relative ordering matters, even small, consistent improvements in ROC-AUC can shift a meaningful number of compounds across a decision threshold and are therefore of practical value.

ADMET-XSpec performs explicit, controllable data-level integration, as compounds are incorporated or excluded based on a user-defined structural similarity threshold, their species or assay origin is retained as an explicit feature, and the resulting models can be built with any standard ML algorithm. On the other hand, transfer and multitask learning rely instead on shared or pretrained representations, typically requiring neural network architectures, which can be similarly effective but are less interpretable. The two approaches are complementary rather than mutually exclusive, and combining attributed, similarity-controlled data selection with transfer learning-style shared representations represents a promising direction for future work.

## Conclusions

The study systematically investigated the impact of cross-species and cross-assay data integration on ML-based ADMET property prediction across four pharmacologically relevant endpoints. Our results demonstrate that the impact of augmentation of training data with pieces of information from different sources depends strongly on different factors: predicted property, data representation, and algorithm. Structured augmentation using explicit source-domain encoding was in general beneficial in terms of models’ predictive power in comparison to naïve data merging (potentially leading to conflicts of labels).

Outcomes obtained in our study underscore that cross-species and cross-assay data integration must be evaluated empirically independently for each case. To this end, we provide ADMET-XSpec, a flexible and open-source computational framework enabling controlled and comprehensive examination of the influence of the integration of heterogeneous data sets. The package is freely available at https://github.com/hubertrybka/admet-xspec.

## Supplementary Material









## Data Availability

The code is made available on GitHub under the MIT license, along with all data sets used for training the model: https://github.com/hubertrybka/admet-xspec

## Notes

ChEMBL IDs of assays used in the study, complete data sets for all experiments, random split all results, and scaffold split all results (ZIP)
Hyperparameters used for model training, dependencies, data set sizes, and an example description of the obtained results (PDF)
Analysis of interspecies consistency of in vitro data (PDF)
Evaluation metrics for all experiments (CSV)

## Abbreviations

AChE: acetylcholinesterase
ADMET: absorption, distribution, metabolism, excretion, and toxicity
BBB: blood–brain barrier
*T*_c_: Tanimoto coefficient
ECFP4: extended-connectivity fingerprint
MACCS: molecular ACCEs system
MAO-A: monoamine oxidase A
ML: machine learning
MSE: mean square error
PAMPA: parallel artificial membrane permeability assay
RF: random forest
SVM: support vector machines
