# Supplementary material for: ADMET-XSpec: A Tool for Systematic Cross-Species Data Integration in ADMET Prediction
Source: Chem Res Toxicol. 2026 Jul 20;39(8):1518–31. doi: 10.1021/acs.chemrestox.5c00562 (PMC13488492; doi:10.1021/acs.chemrestox.5c00562)
Supplement: Supplementary file 1 [file tx5c00562_si_002.pdf]

SVM

C ~ LogUniform(0.1, 1000)
gamma ~ LogUniform(0.001, 1)

RF

n_estimators ~ QLogUniform(100, 2000)
max_depth ~ QUniform(2, 100)
min_samples_split ~ QUniform(2, 10)
min_samples_leaf ~ QUniform(1, 10)

LGBM

n_estimators ~ QLogUniform(100, 2000)
max_depth ~ QUniform(2, 50)
num_leaves ~ QUniform(20, 200)
min_child_samples ~ QUniform(10, 100)

where Uniform(a, b), LogUniform(a, b) denote contiuous uniform and log-uniform distributions over the range of [a, b], and QUniform(a, b), QlogUniform(a, b) denote discreete uniform and log-uniform distributions over integers in the range of [a, b]

**Dependencies:**

lightgbm==4.6.0
matplotlib==3.10.7
numpy==2.3.3
optuna==4.8.0
rdkit==2025.3.6
scikit_learn==1.7.2
scipy==1.16.2
umap-learn>0.5.8

**Number of compounds present in each dataset upon application of the splitting strategy described in the paper**

| **Dataset** | **Task** | **Train** | **Test** | **% Train** | **% Test** |
|---|---|---|---|---|---|
| AChE | regression | 6017 | 1504 | 80.00% | 20.00% |
| AChE | binary classification | 6042 | 1510 | 80.01% | 19.99% |
| MAOA | regression | 2565 | 641 | 80.01% | 19.99% |
| MAOA | binary classification | 2593 | 648 | 80.01% | 19.99% |
| Liver t_1/2 | regression | 3181 | 795 | 80.01% | 19.99% |
| Liver t_1/2 | binary classification | 3319 | 829 | 80.01% | 19.99% |
| BBB Permeability | binary classification | 6243 | 1560 | 80.01% | 19.99% |

## Example results description

### $T_{1/2}$ liver (LightGBM)

The impact of cross-species data integration for LightGBM was strongly endpoint-, representation-, and threshold-dependent, and augmentation was not universally beneficial (Figure S1-S3). A consistent finding across all representations for LightGBM was that compounds with Tanimoto distance ≥ 0.6 to the human training set contributed negligibly to model performance, (differences below 0.01 for AUC-ROC and 0.05 in terms of MSE, relative to the human-only baseline regardless of augmentation strategy). At Tc distance = 0.0 (where structurally identical cross-species compounds are present) the choice of augmentation strategy was critical. The effect of naïve

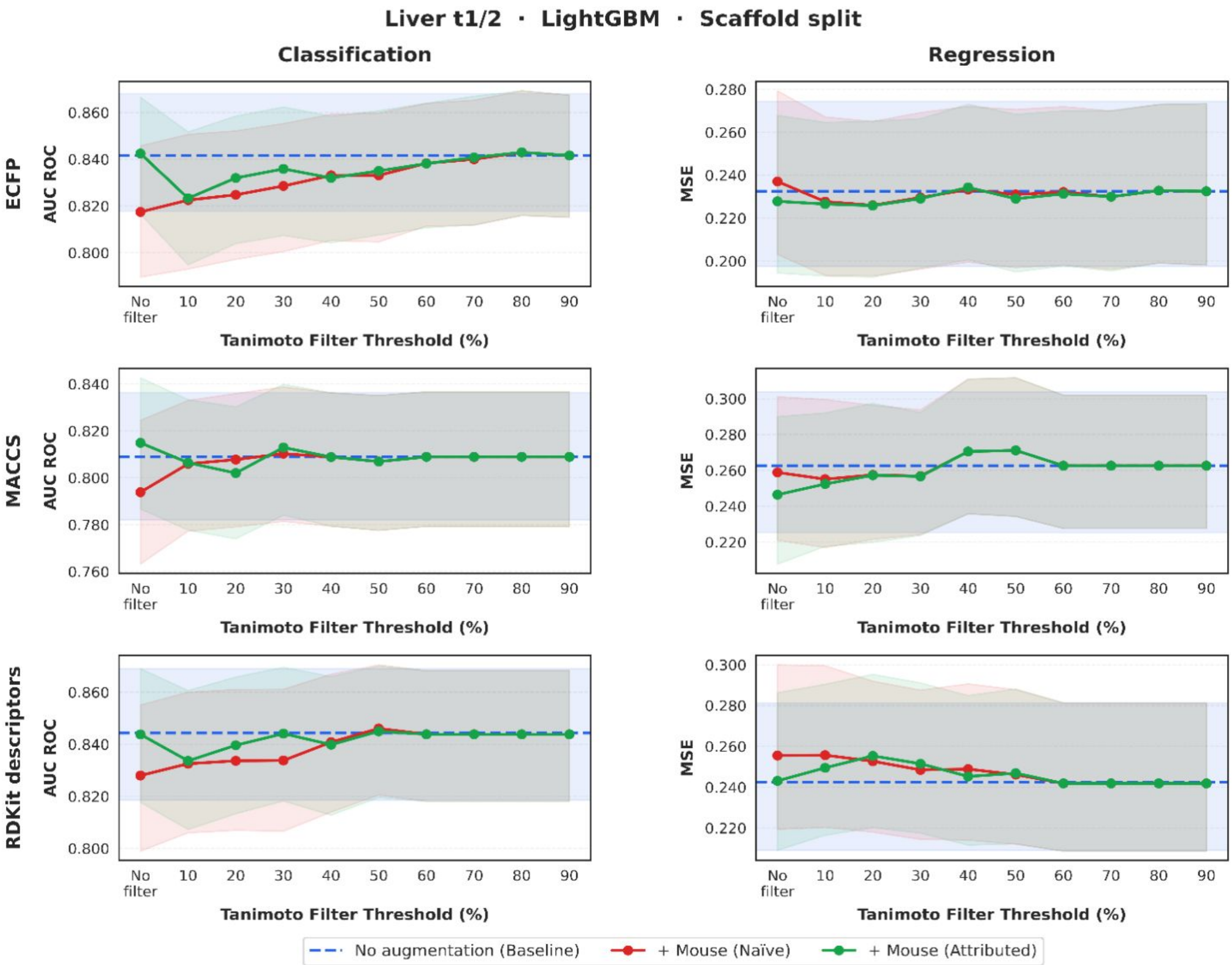


**Figure S1**. Analysis of the influence of human-data-based training set augmentation with mouse data on the LightGBM-based models performance predicting $t_{1/2}$ liver. Shaded areas represent 95% bootstrap confidence intervals.

augmentation on models predictive power was not uniform across representations and species combinations.  For ECFP-based and RDKit Descriptors-based models, naïve augmentation with rat data increased MSE by approximately 0.04, whereas for MACCSFP, the MSE was reduced by ~0.03 upon analogous augmentation. When additional mouse data were incorporated (rat + mouse scenario), ECFP-based models showed MSE values comparable to those obtained using human data only. In contrast, MACCS fingerprints resulted in a decrease in MSE of approximately 0.04, whereas RDKit descriptors exhibited an increase of ~0.02. Similar trends were observed for classification models, with naïve augmentation with rat (and subsequently rat + mouse) data, leading to AUC-ROC drops of approximately 0.1 and 0.2 for ECFP- and RDKit Descriptors-based models, respectively, and an ROC-AUC improvement of ~0.01 for MACCS fingerprint-based models.

Attributed augmentation for lower Tanimoto thresholds (Tc = 0.1-0.4) yielded modest MSE reductions (~0.01–0.02) for addition of mouse data, but simultaneously a slight drop in ROC-AUC, most pronounced at Tc = 0.1 (~0.02 for ECFP and RDKit descriptors). For augumentation of rat data, ROC-AUC was substantially higher for MACCS fingerprints (by up to ~0.02) but marginally lower for other representations (by 0.005–0.01) in classification experiments. When regression is considered, MSE was also higher for MACCS fingerprints in this scenario (up to 0.01 at Tc = 0.3), while for ECFP and RDKit descriptors the influence on model performance was negligible (MSE change ≤ 0.002). For combined rat+mouse augmentation, attributed augmentation reduced ROC-AUC by up to 0.01–0.02, with generally larger effects for ECFP than RDKit descriptors and slightly

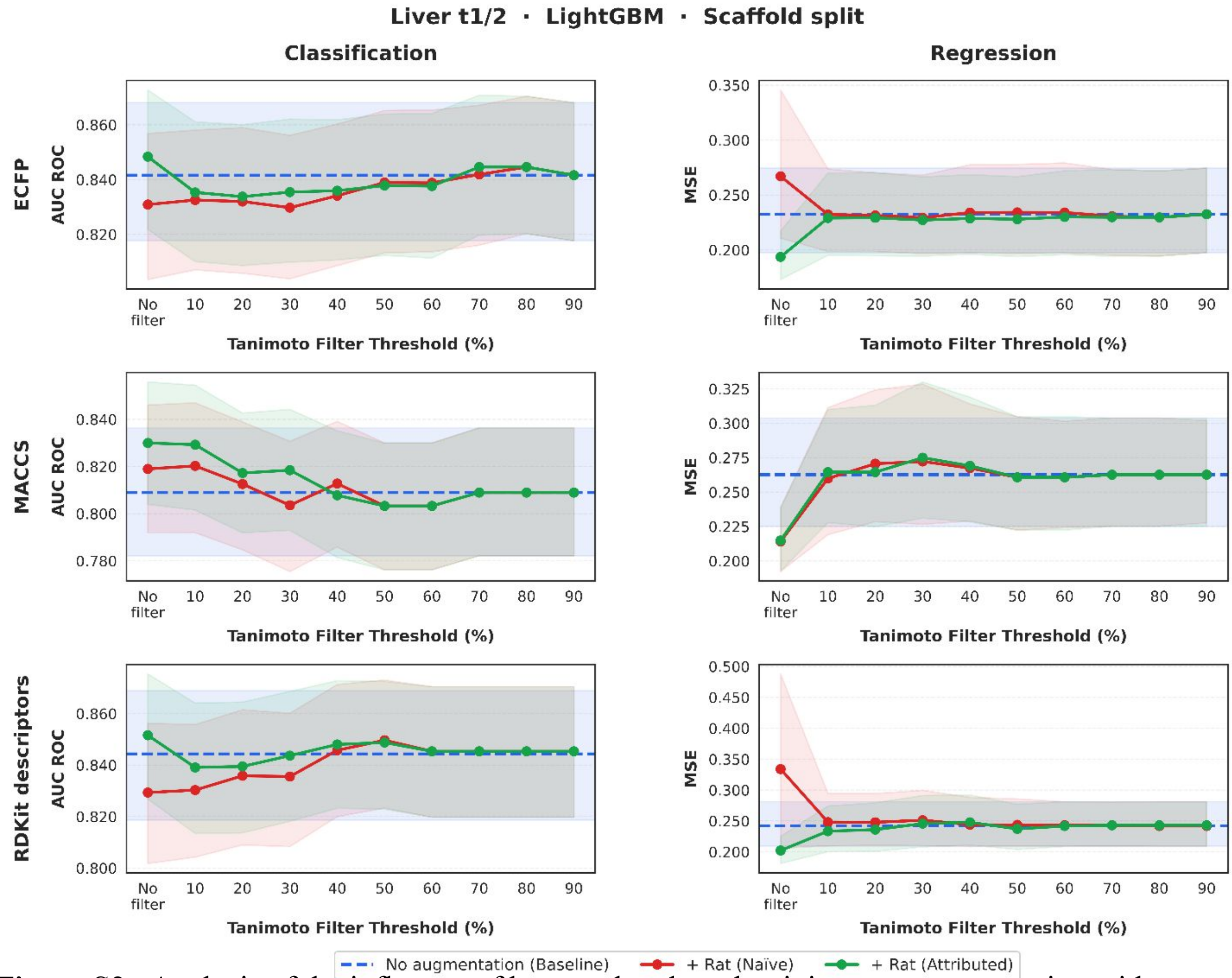


**Figure S2**. Analysis of the influence of human-data-based training set augmentation with rat data on the LightGBM-based models performance predicting $t_{1/2}$ liver. Shaded areas represent 95% bootstrap confidence intervals.

smaller effects for MACCS fingerprints (~0.005). MSE reductions were also observed: ~0.015 for

MACCS fingerprints and ECFP, whereas RDKit descriptor-based models showed increased MSE (~0.1–0.15).

Overall, the Tanimoto similarity of incorporated compounds was a stronger determinant of model performance than the augmentation strategy employed, underscoring the primacy of chemical space considerations in cross-species data integration in this particular scenario.

**$T_{1/2}$ liver (SVM)**

SVM models exhibited the greatest sensitivity to training data modifications at Tc = 0.0 (Figure S4, Supporting Information, ZIP File). In classification, attributed augmentation consistently outperformed baseline and naïve augmentation across all representations: ECFP-based models exceeded baseline (AUC-ROC ~0.840 vs. ~0.830), while naïve augmentation started below baseline

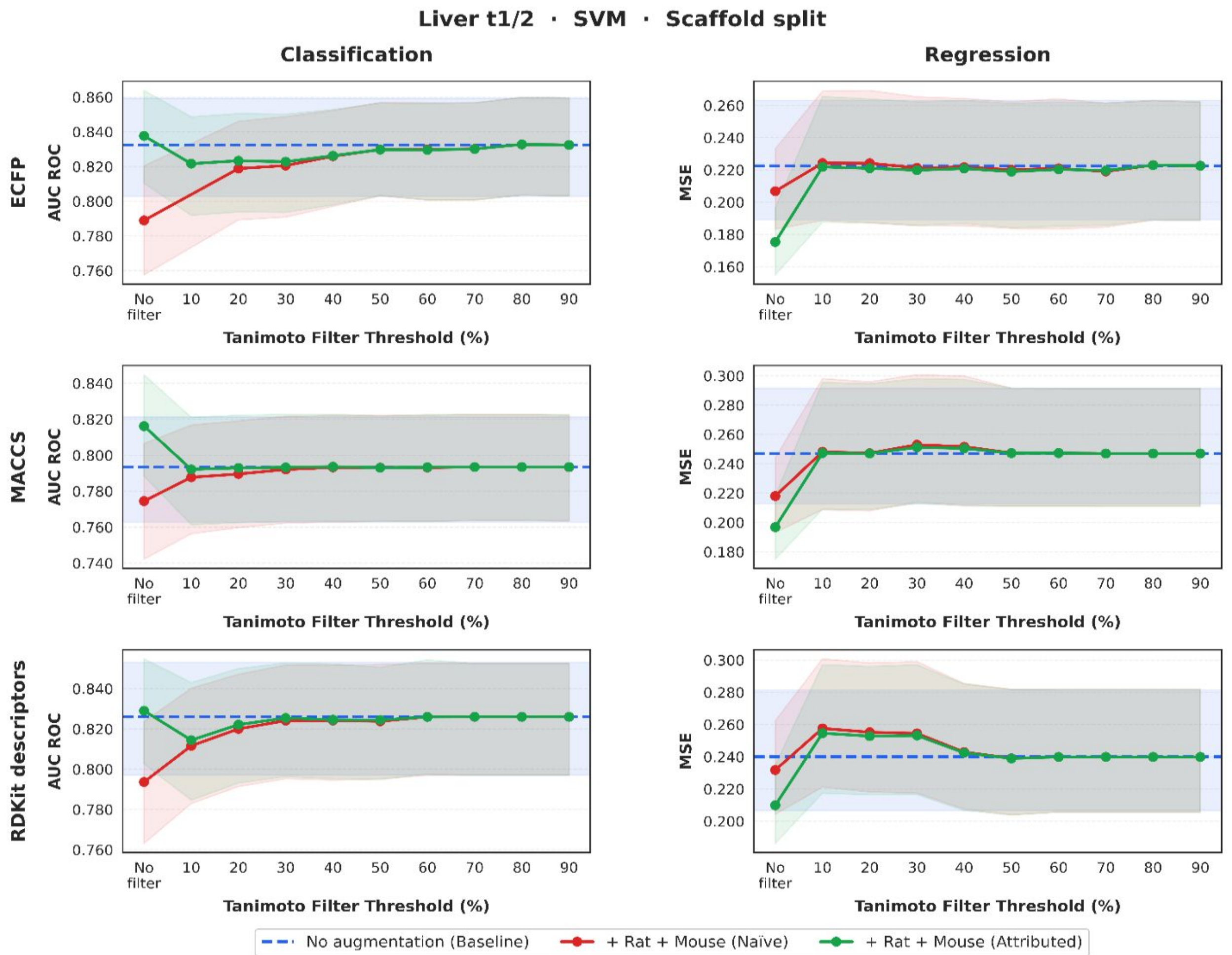


**Figure S3**. Analysis of the influence of human-data-based training set augumentation with rat and mouse data on the LightGBM-based models performance predicting $t_{1/2}$ liver. Shaded areas represent 95% bootstrap confidence intervals.

**Figure S4**. Analysis of the influence of human-data-based training set augumentation with rat and mouse data on the SVM-based models performance predicting $t_{1/2}$ liver. Shaded areas represent 95% bootstrap confidence intervals.

(~0.780), only approaching it at Tc = 0.40–0.50. MACCS and RDKit models showed similar trends, with attributed augmentation at or above baseline and naïve augmentation remaining below baseline throughout most of the Tc range. In regression, attributed augmentation consistently yielded lower MSE than naïve augmentation across all representations (e.g. ECFP: ~0.175 vs. ~0.205 forc Tc = 0.0, with both strategies converging toward the human-only baseline (~0.220) at Tc ≥ 0.10; MACCSFP: ~0.215 vs. ~0.235 for Tc = 0.0 with baseline equal to 0.240; reaching values above baseline (~0.250-0.260 for Tc between 0.1 and 0.3). Above Tc = 0.5, augmentation had no measurable impact on SVM regression performance for both regression and classification tasks.

**$T_{1/2}$ liver (RF)**

For RF models, the augmentation strategy was the primary determinant of the direction of performance change (Figure S5, Supporting Information, ZIP File). For ECFP and MACCS fingerprints, incorporation of rat or combined rat + mouse data via attributed augmentation improved classification ROC-AUC by ~0.01 relative to the human-only baseline, whereas naïve augmentation degraded performance by a comparable magnitude (~0.01). This effect was most pronounced at Tc = 0.0 and diminished progressively, converging to baseline performance at the added compounds distance of Tc ≈ 0.3. In regression (rat + mouse data; Tc = 0.0), ECFP-based models favoured

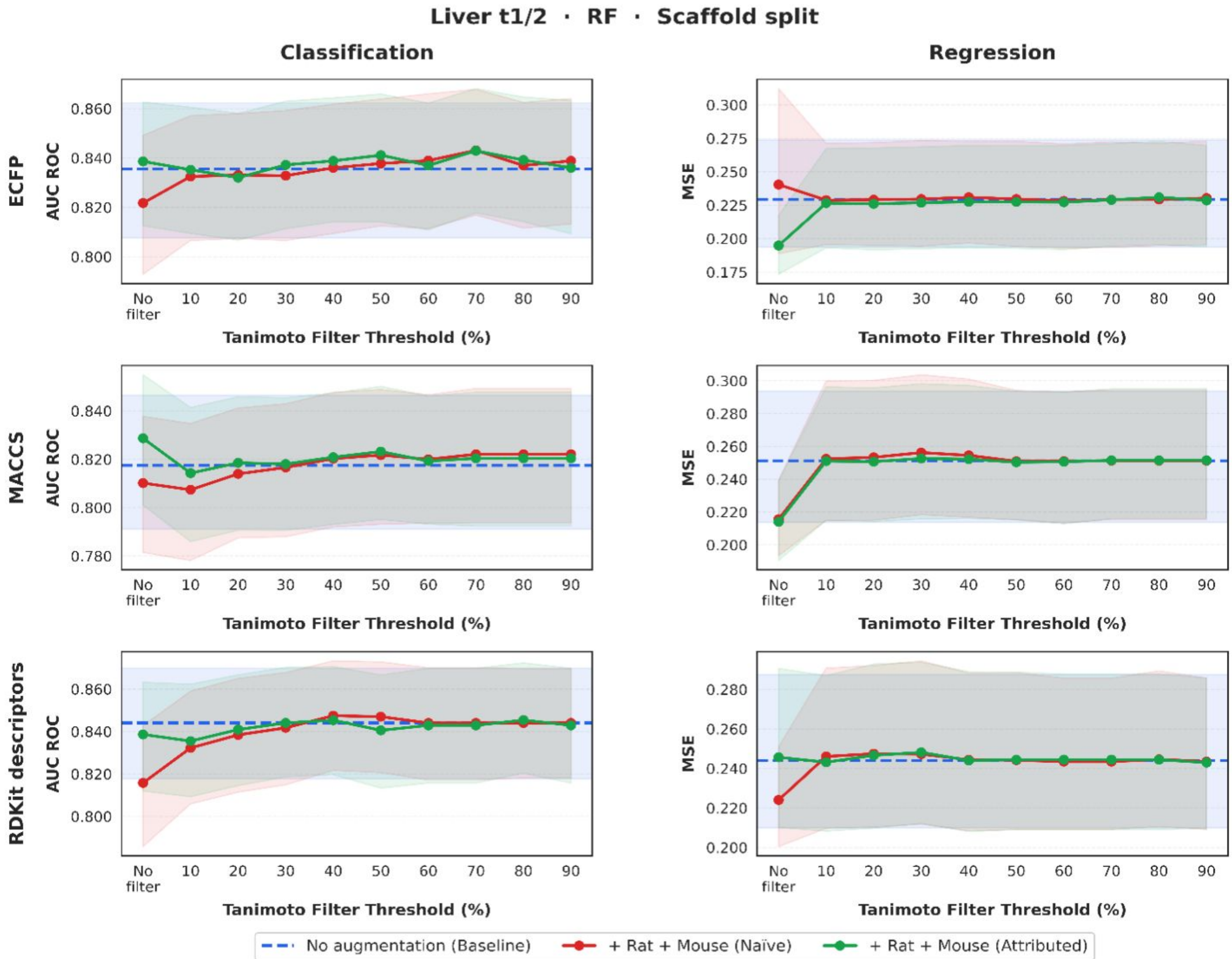


**Figure S5**. Analysis of the influence of human-data-based training set augumentation with rat and mouse data on the RF-based models performance predicting $t_{1/2}$ liver. Shaded areas represent 95% bootstrap confidence intervals.

attributed augmentation, models based on MACCSFP benefited from both strategies, while RDKit descriptor-based models showed lower MSE under naïve augmentation. For higher Tc values, differences between strategies diminished and MSE converged toward the human-only baseline.

Mouse-only augmentation showed a distinct pattern: ROC-AUC was reduced across all strategies for Tc $\leq 0.3$, with only marginal improvements ($<0.005$) observed for Tc $> 0.5$. Regression performance was similarly unaffected, with the exception of Tc = 0.0 where a negligible change in MSE ($<0.005$) was noted.
