# Supplementary material for: ADMET-XSpec: A Tool for Systematic Cross-Species Data Integration in ADMET Prediction
Source: Chem Res Toxicol. 2026 Jul 20;39(8):1518–31. doi: 10.1021/acs.chemrestox.5c00562 (PMC13488492; doi:10.1021/acs.chemrestox.5c00562)

AChE

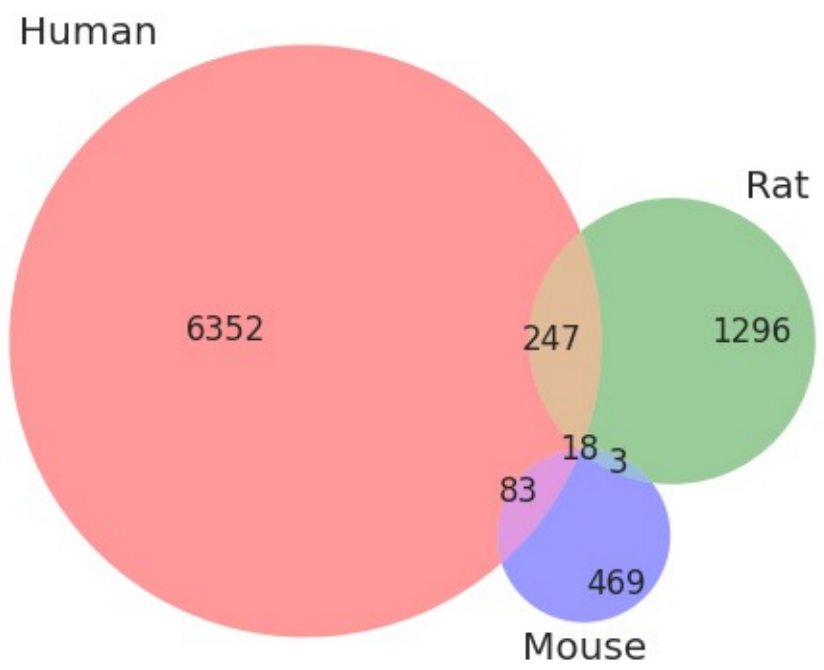

Classification

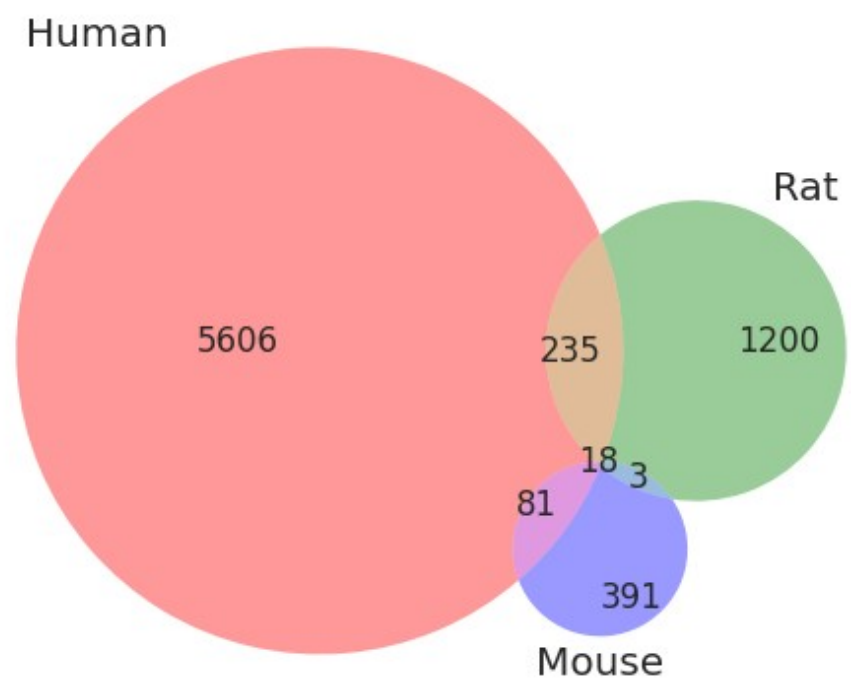

Regression

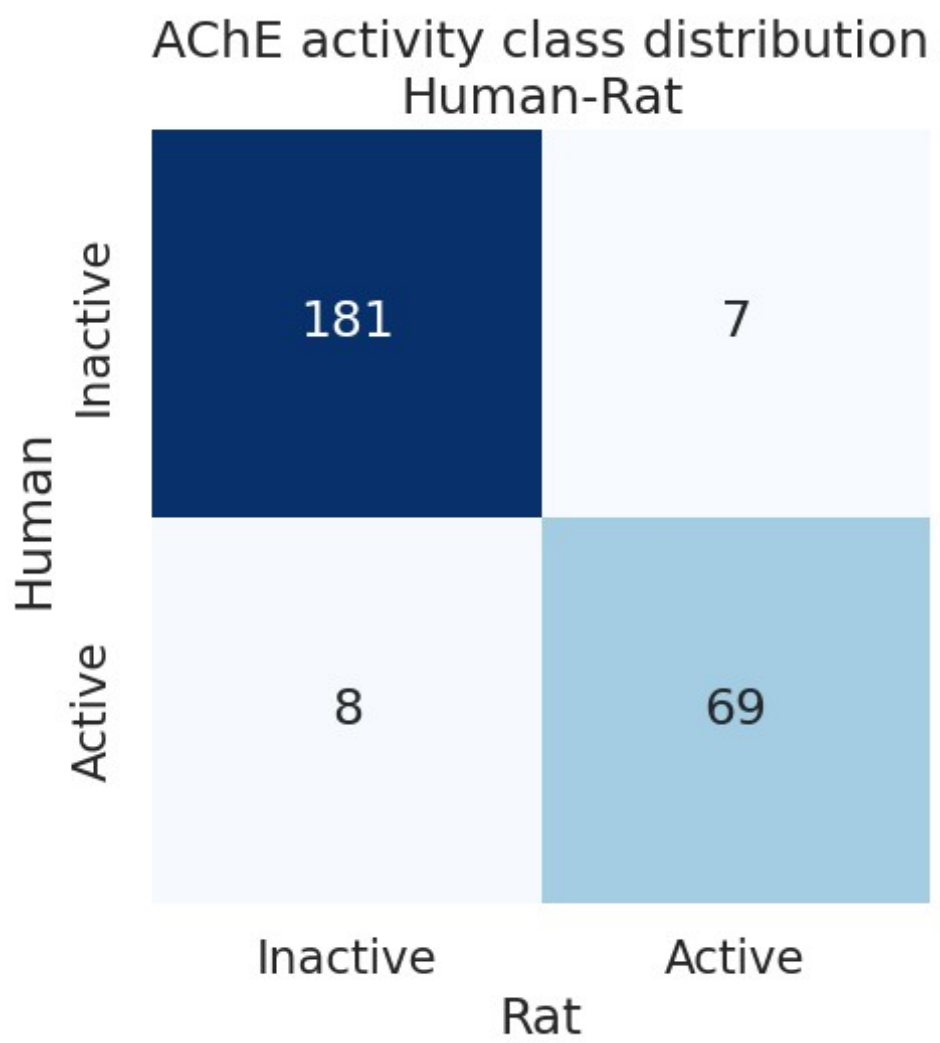

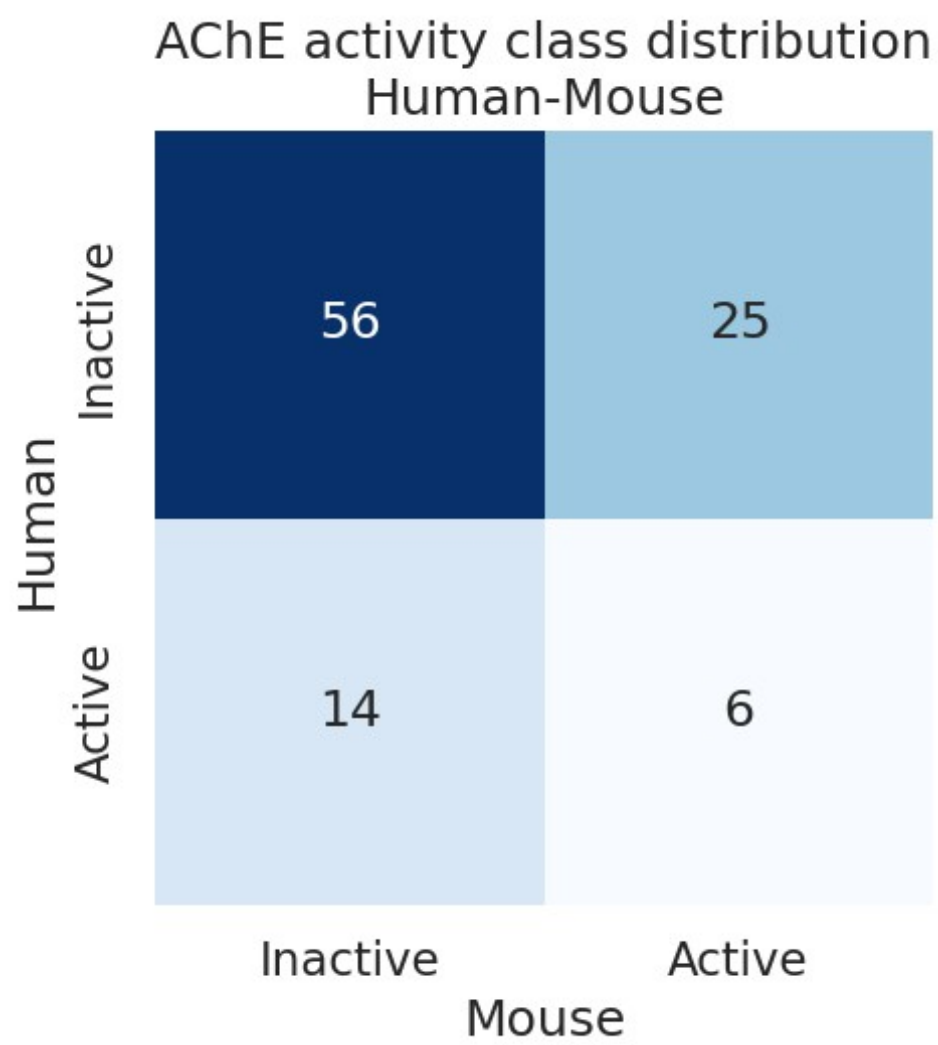

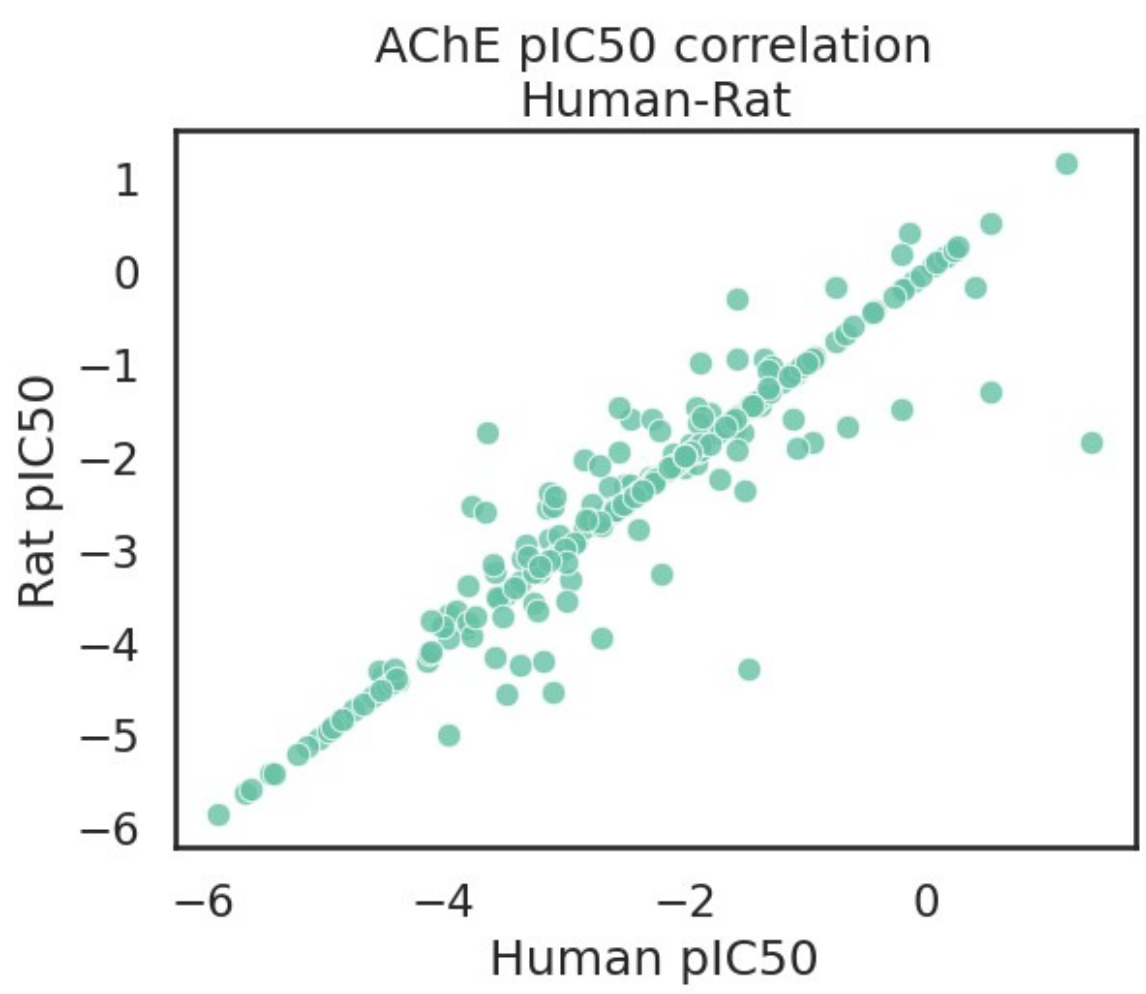

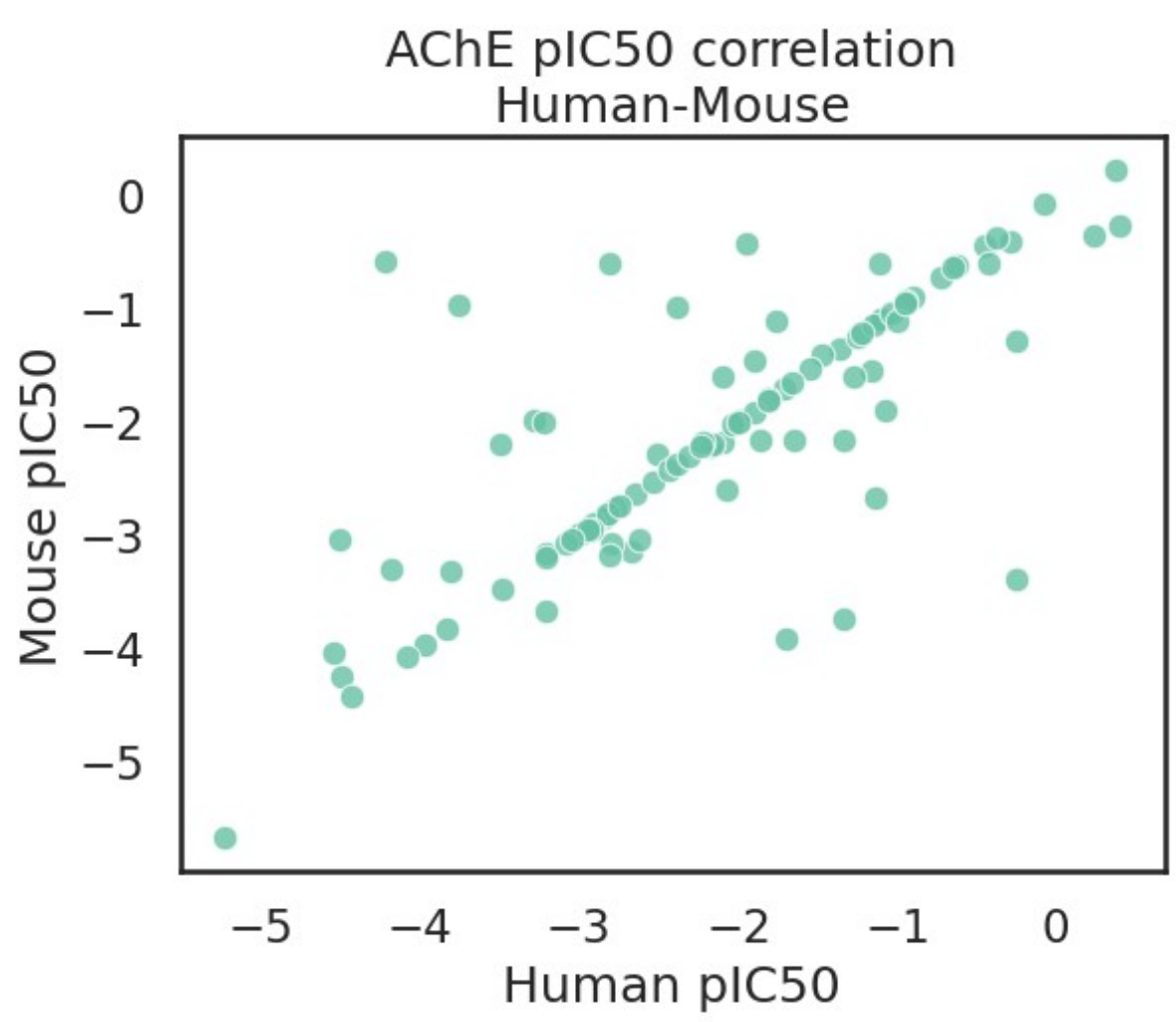

MAO-A

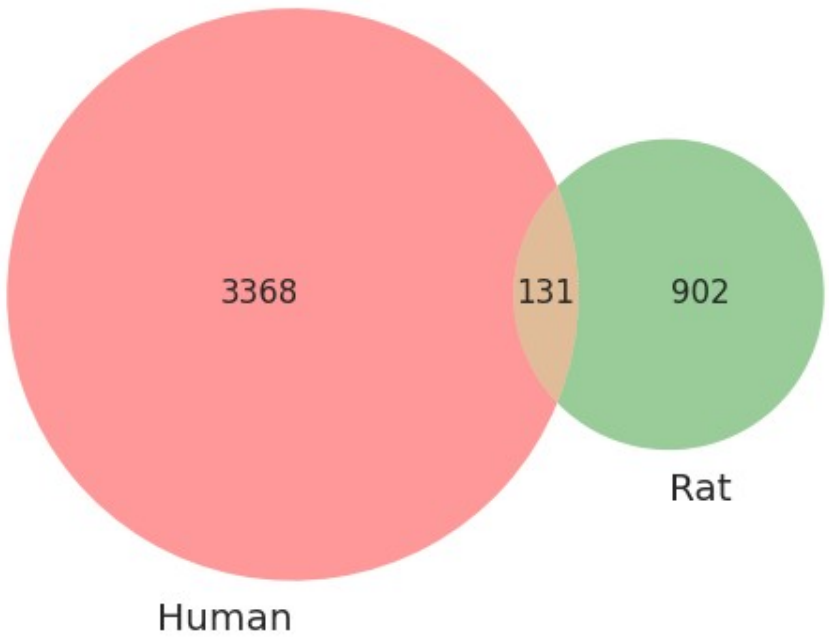

Classification

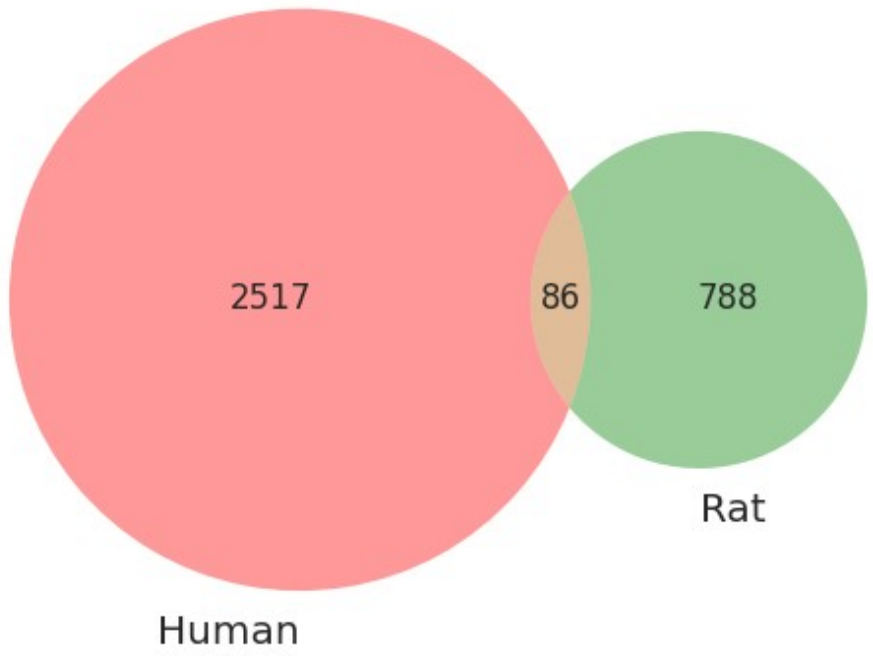

Regression

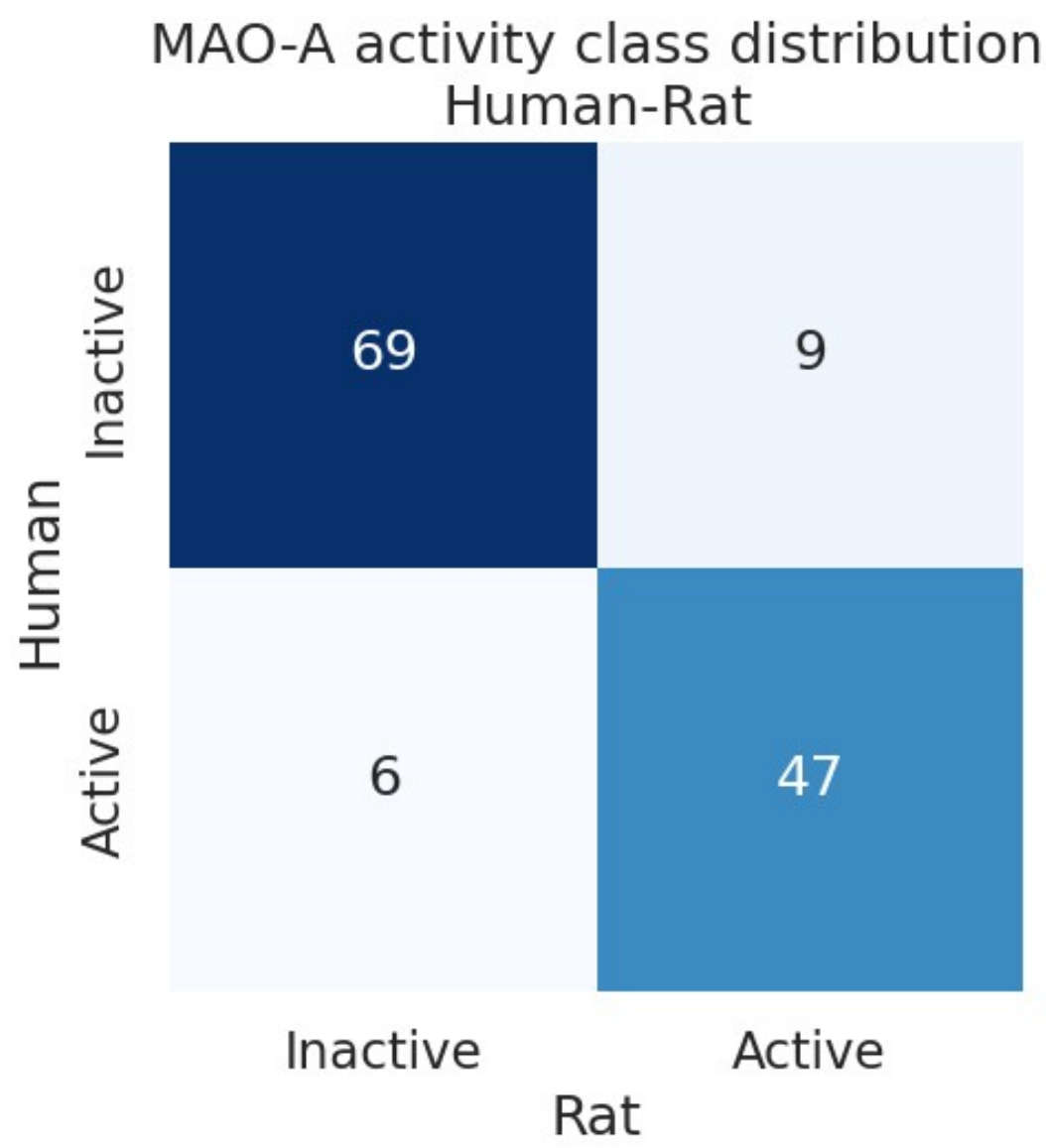

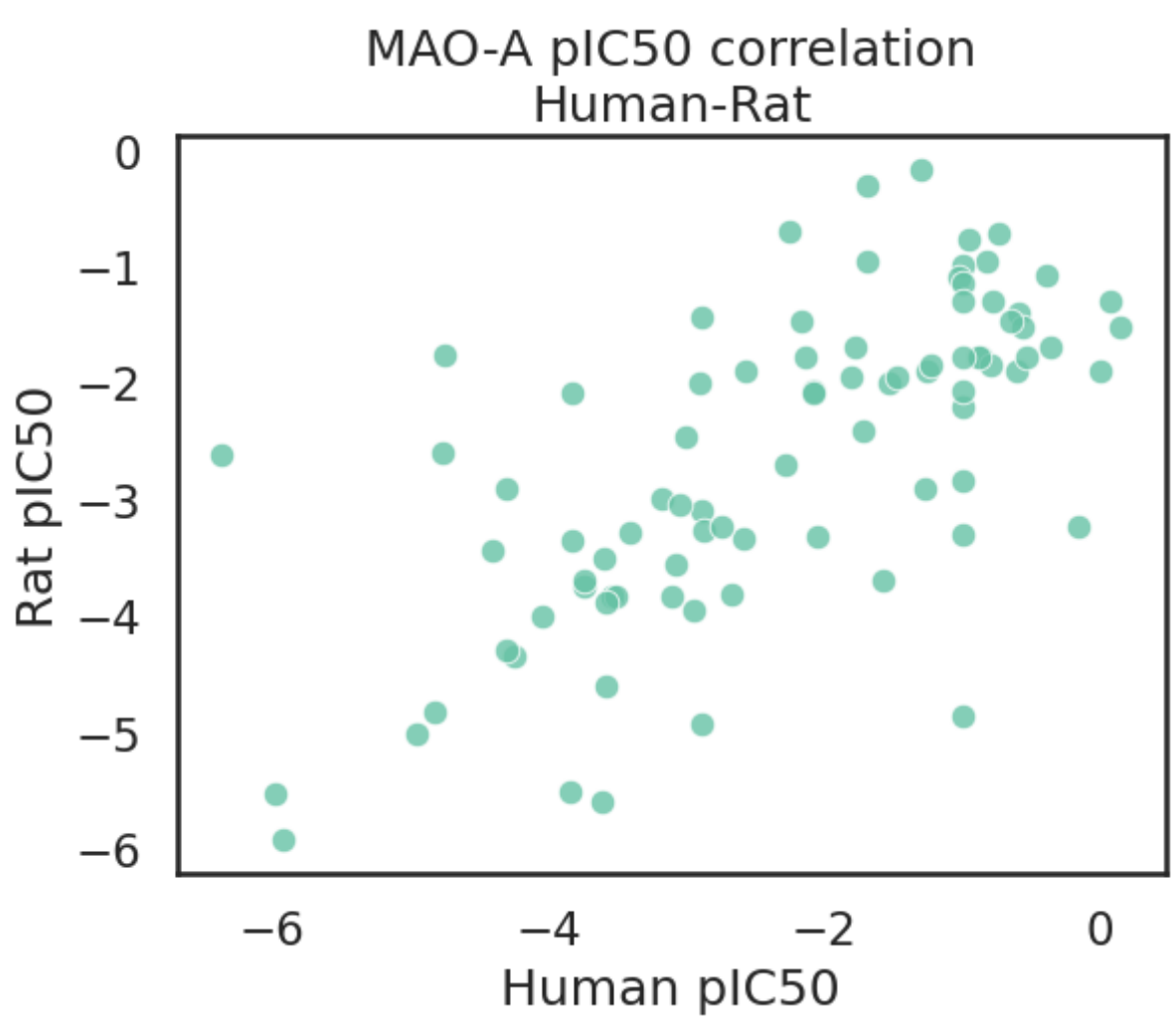

Liver t ½

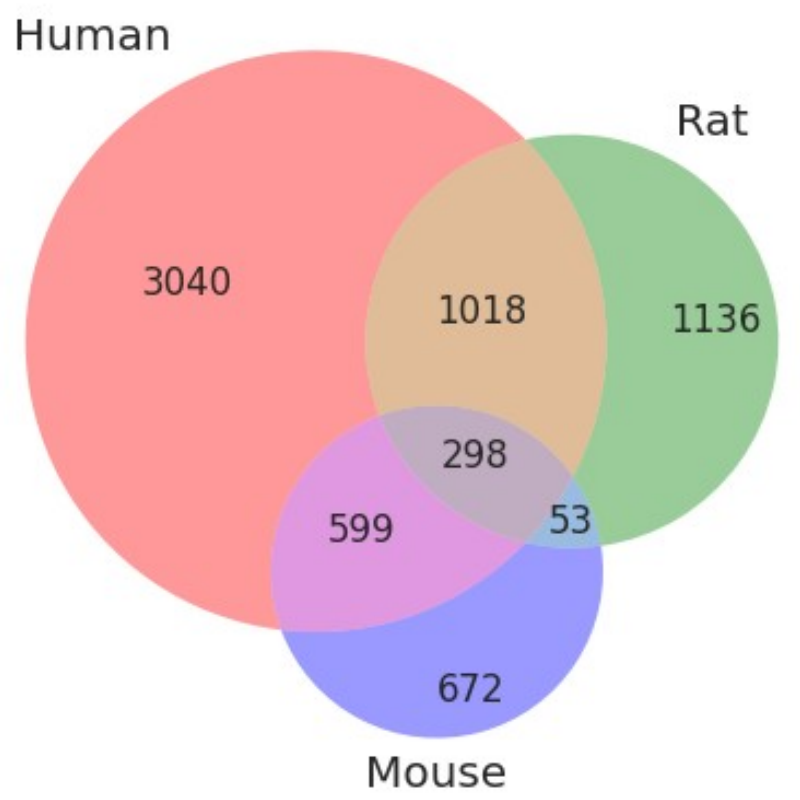

Classification

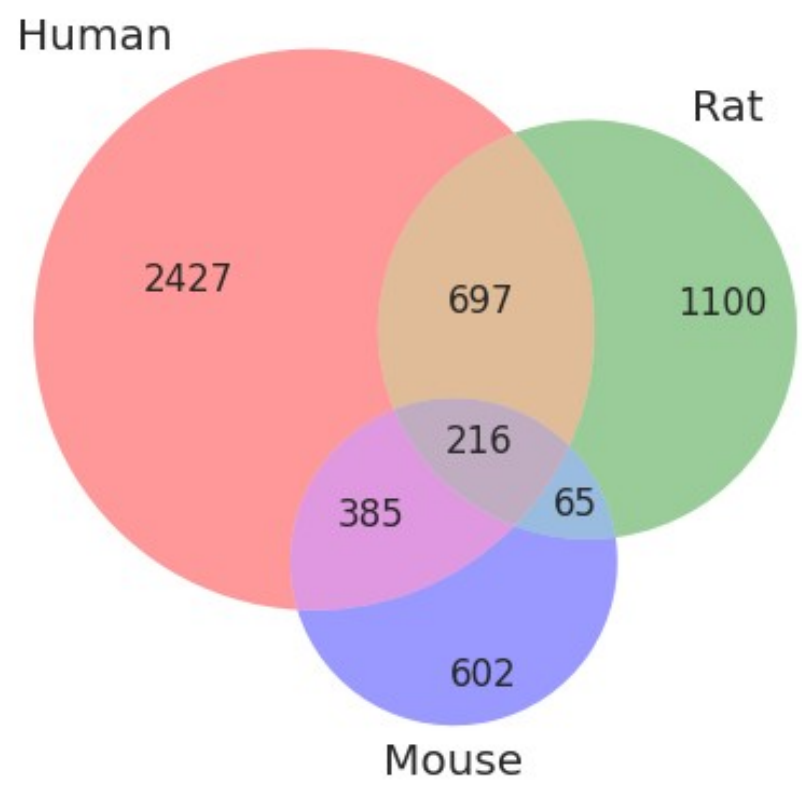

Regression

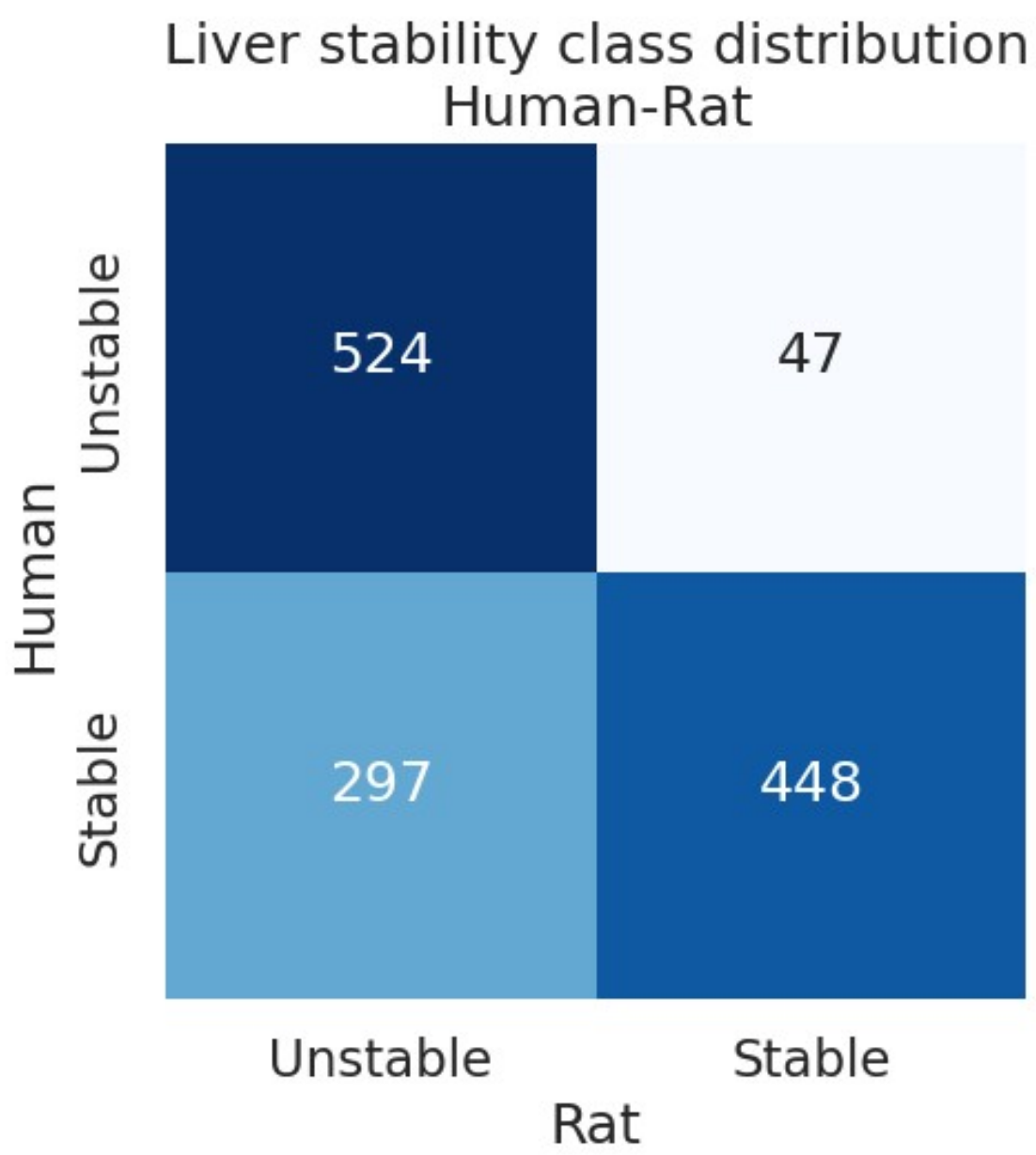

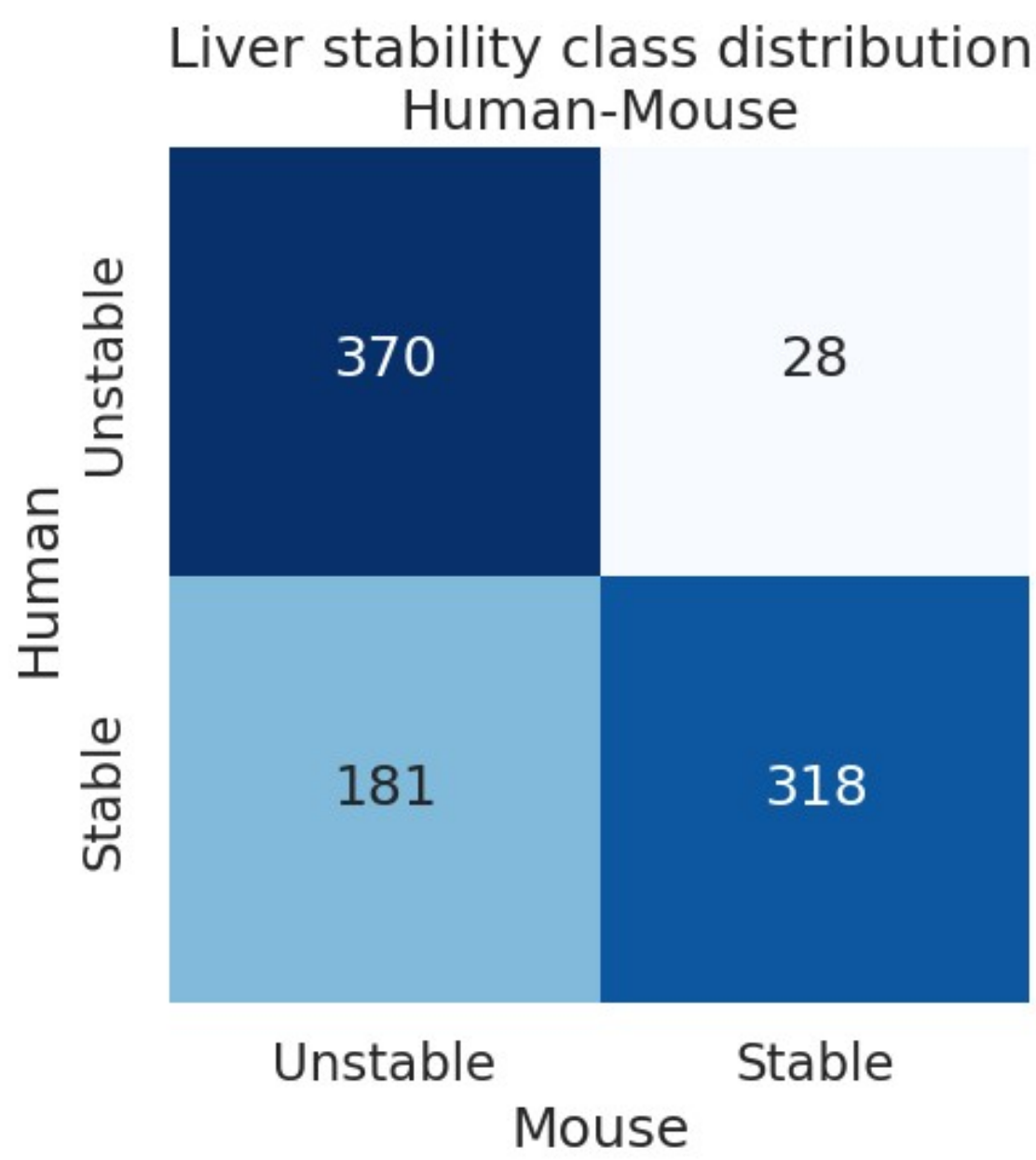

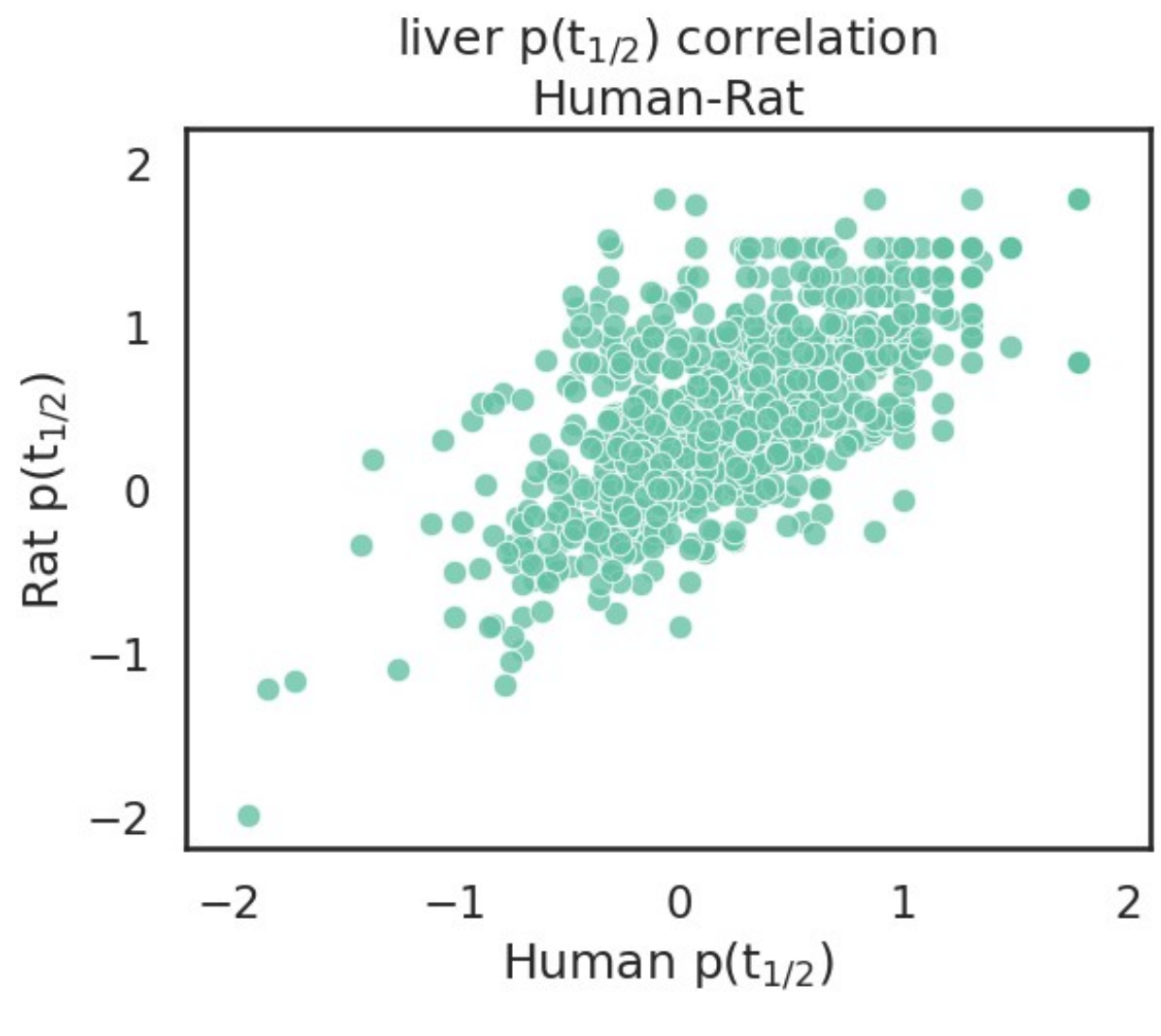

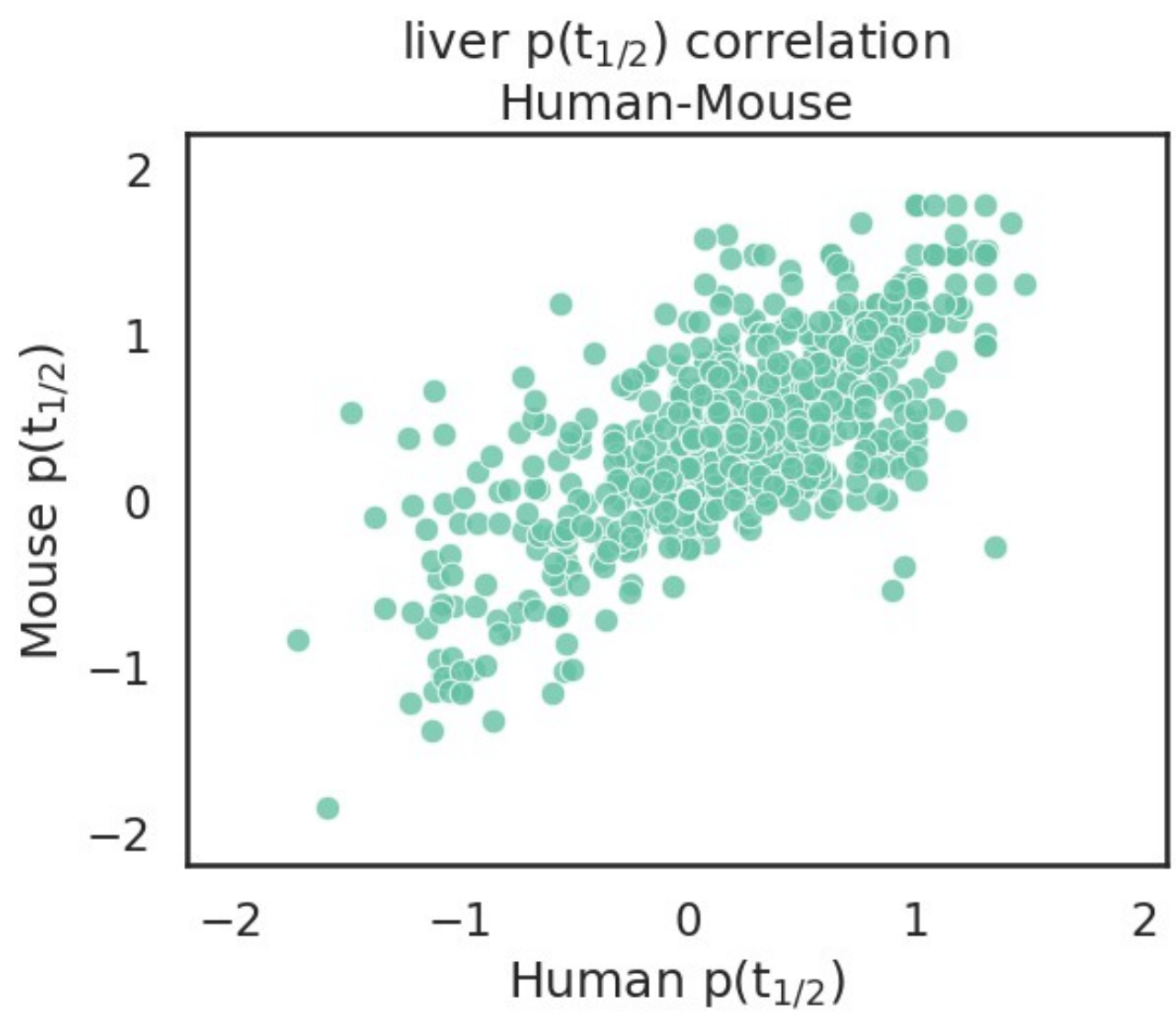

Permeability

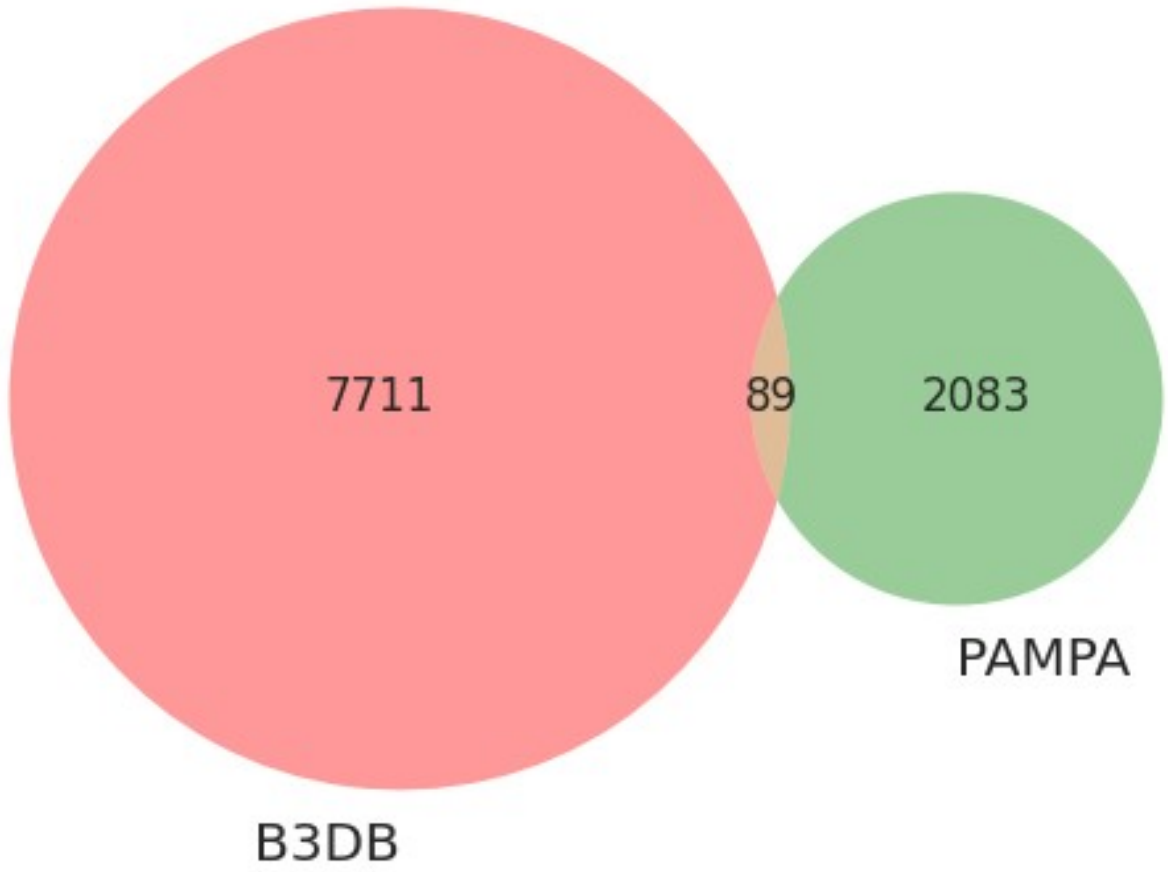

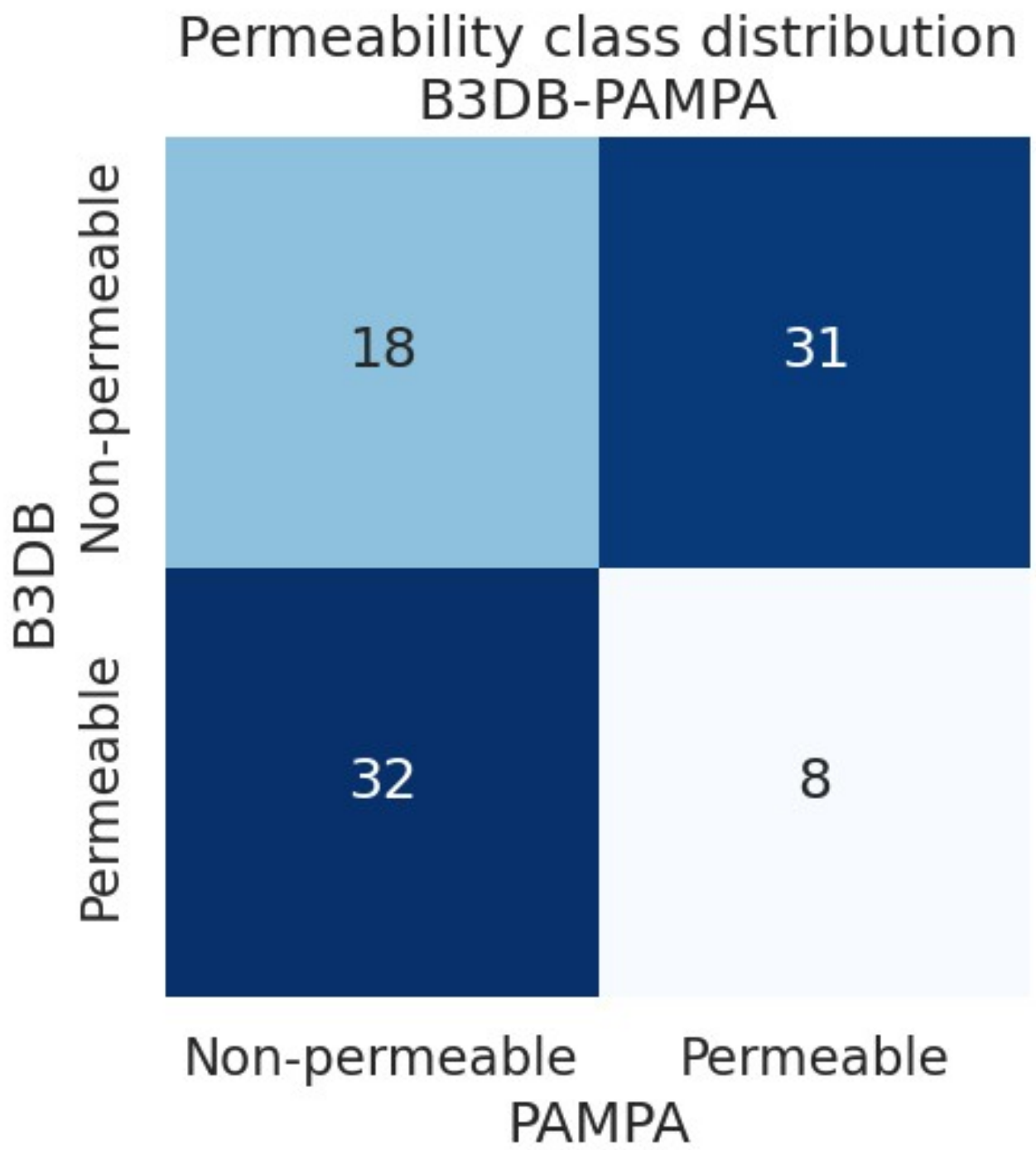

Supplement: Supplementary file 2 [file tx5c00562_si_003.pdf]
