# Supplementary material for: ADMET-XSpec: A Tool for Systematic Cross-Species Data Integration in ADMET Prediction
Source: Chem Res Toxicol. 2026 Jul 20;39(8):1518–31. doi: 10.1021/acs.chemrestox.5c00562 (PMC13488492; doi:10.1021/acs.chemrestox.5c00562)
Supplement: Supplementary file 3 [file tx5c00562_si_001.zip › datasets_assay_IDs_random_and_scaffold_split_complete_results/random_split_results/AChE/6panel__AChE_human_pIC50__LightGBM__Random__Rat.pdf]

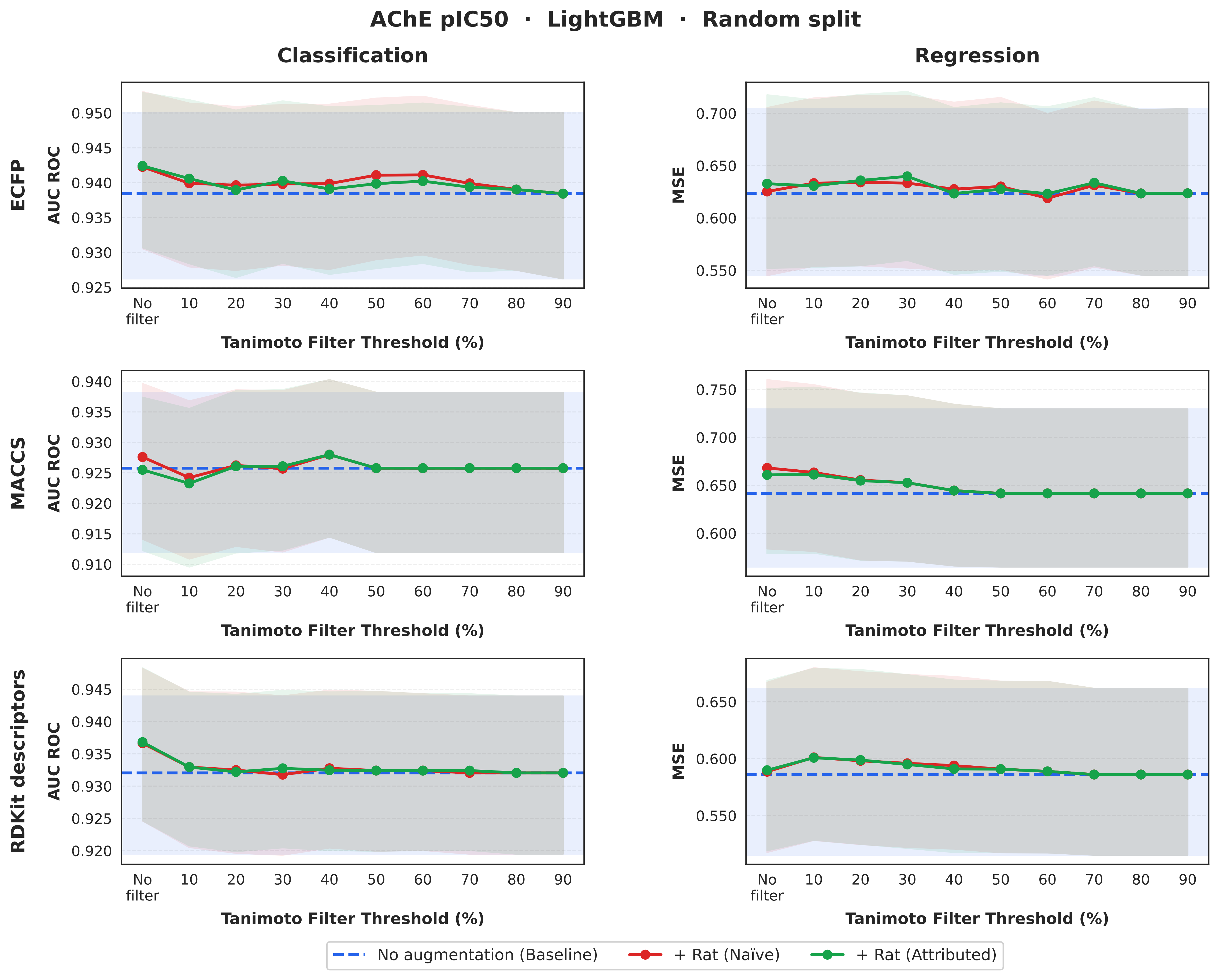
AChE pIC50 · LightGBM · Random split
Classification
Regression
ECFP
MACCS
RDKit descriptors
AUC ROC
MSE
Tanimoto Filter Threshold (%)
No filter
10
20
30
40
50
60
70
80
90
No augmentation (Baseline)
+ Rat (Naïve)
+ Rat (Attributed)
