# Supplementary material for: ADMET-XSpec: A Tool for Systematic Cross-Species Data Integration in ADMET Prediction
Source: Chem Res Toxicol. 2026 Jul 20;39(8):1518–31. doi: 10.1021/acs.chemrestox.5c00562 (PMC13488492; doi:10.1021/acs.chemrestox.5c00562)
Supplement: Supplementary file 3 [file tx5c00562_si_001.zip › datasets_assay_IDs_random_and_scaffold_split_complete_results/random_split_results/Liver/6panel__liver_human_pT12__LightGBM__Random__Mouse.pdf]

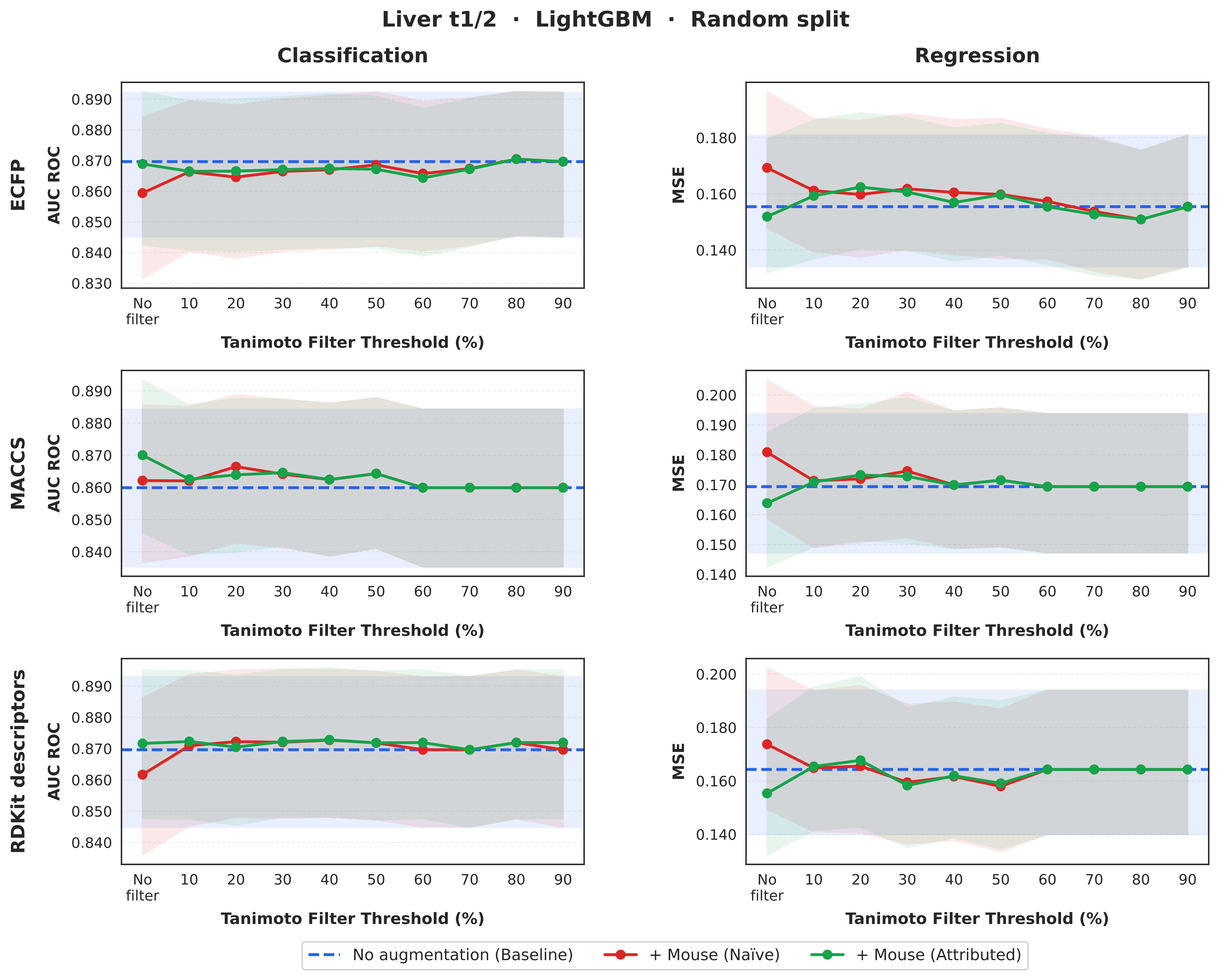
Liver t1/2 · LightGBM · Random split
Classification
Regression
ECFP
MACCS
RDKit descriptors
AUC ROC
MSE
Tanimoto Filter Threshold (%)
No filter
10
20
30
40
50
60
70
80
90
0.830
0.840
0.850
0.860
0.870
0.880
0.890
0.140
0.150
0.160
0.170
0.180
0.190
0.200
No augmentation (Baseline)
+ Mouse (Naïve)
+ Mouse (Attributed)
