# Supplementary material for: ADMET-XSpec: A Tool for Systematic Cross-Species Data Integration in ADMET Prediction
Source: Chem Res Toxicol. 2026 Jul 20;39(8):1518–31. doi: 10.1021/acs.chemrestox.5c00562 (PMC13488492; doi:10.1021/acs.chemrestox.5c00562)
Supplement: Supplementary file 3 [file tx5c00562_si_001.zip › datasets_assay_IDs_random_and_scaffold_split_complete_results/scaffold_split_results/AChE/6panel__AChE_human_pIC50__SVM__Scaffold__Rat.pdf]

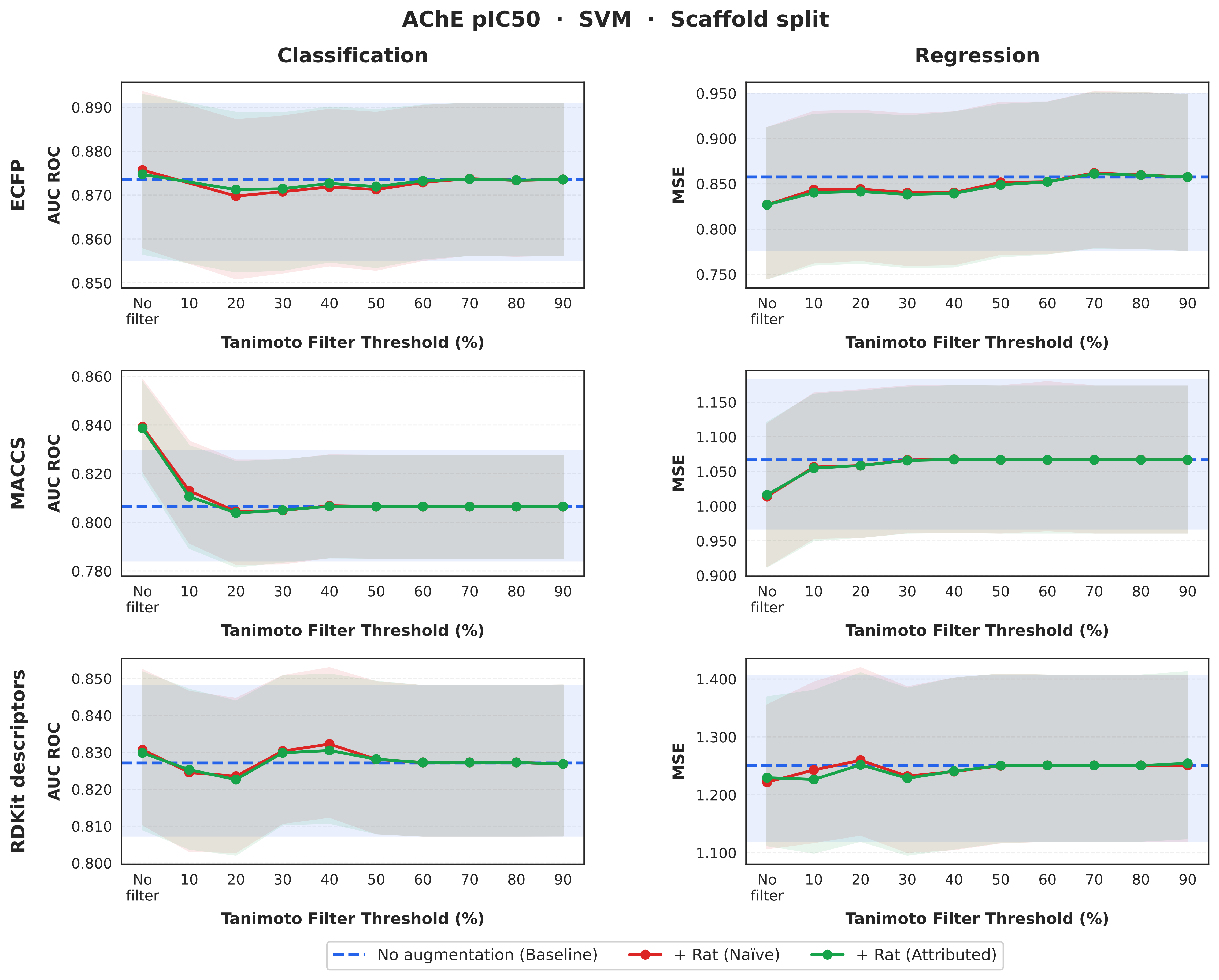
AChE pIC50 · SVM · Scaffold split
Classification
Regression
ECFP
MACCS
RDKit descriptors
AUC ROC
MSE
Tanimoto Filter Threshold (%)
No filter
10
20
30
40
50
60
70
80
90
0.890
0.880
0.870
0.860
0.850
0.950
0.900
0.850
0.800
0.750
0.860
0.840
0.820
0.800
0.780
1.150
1.100
1.050
1.000
0.950
0.900
0.850
0.840
0.830
0.820
0.810
0.800
1.400
1.300
1.200
1.100
No augmentation (Baseline)
+ Rat (Naïve)
+ Rat (Attributed)
