# Supplementary material for: ADMET-XSpec: A Tool for Systematic Cross-Species Data Integration in ADMET Prediction
Source: Chem Res Toxicol. 2026 Jul 20;39(8):1518–31. doi: 10.1021/acs.chemrestox.5c00562 (PMC13488492; doi:10.1021/acs.chemrestox.5c00562)
Supplement: Supplementary file 3 [file tx5c00562_si_001.zip › datasets_assay_IDs_random_and_scaffold_split_complete_results/scaffold_split_results/MAOA/6panel__MAOA_human_pIC50__SVM__Scaffold__Rat.pdf]

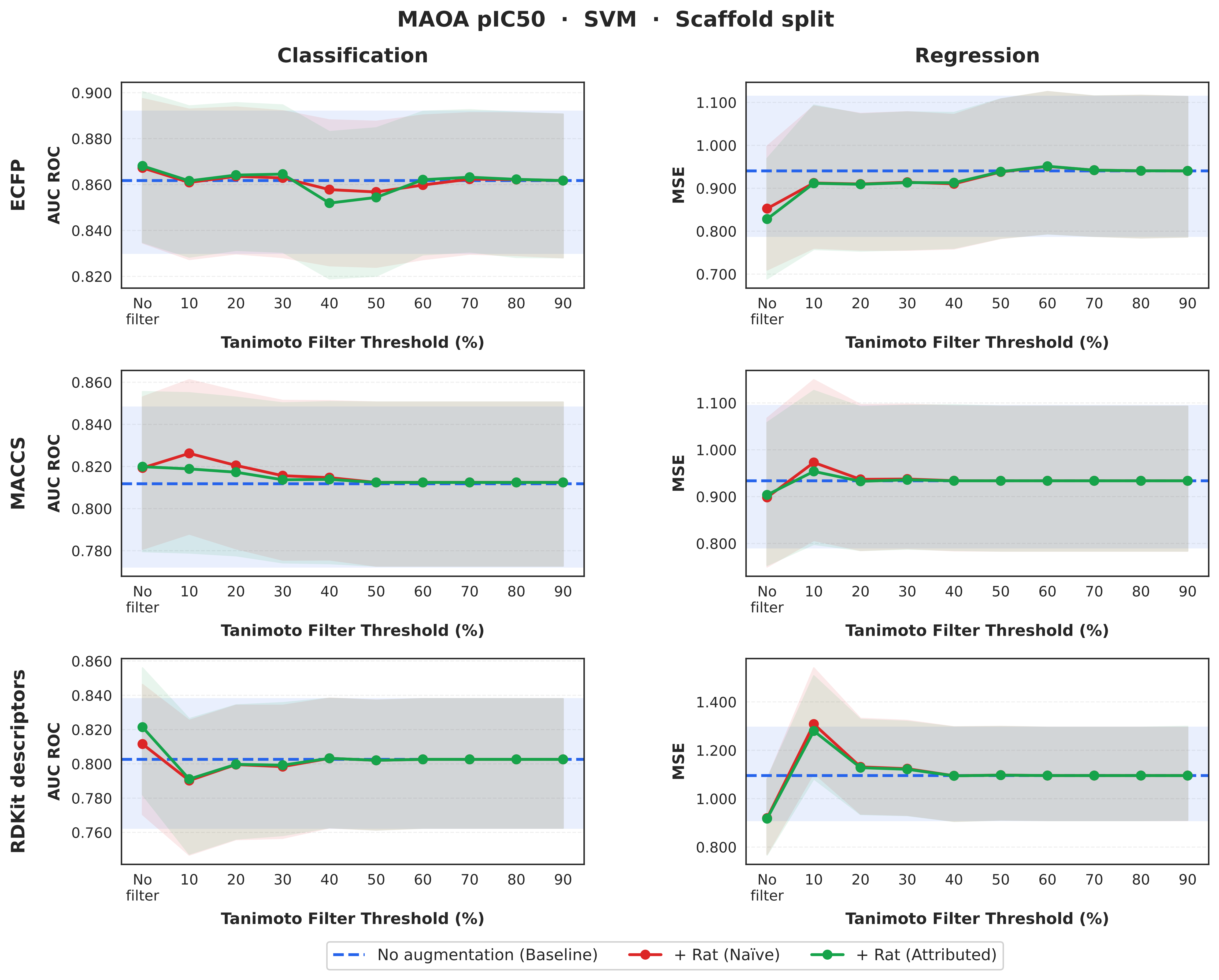

MAOA pIC50 · SVM · Scaffold split
Classification
Regression
ECFP
MACCS
RDKit descriptors
AUC ROC
MSE
Tanimoto Filter Threshold (%)
No filter
10
20
30
40
50
60
70
80
90
0.900
0.880
0.860
0.840
0.820
1.100
1.000
0.900
0.800
0.700
0.780
0.760
1.400
1.200
No augmentation (Baseline)
+ Rat (Naïve)
+ Rat (Attributed)
