# Supplementary material for: ADMET-XSpec: A Tool for Systematic Cross-Species Data Integration in ADMET Prediction
Source: Chem Res Toxicol. 2026 Jul 20;39(8):1518–31. doi: 10.1021/acs.chemrestox.5c00562 (PMC13488492; doi:10.1021/acs.chemrestox.5c00562)
Supplement: Supplementary file 3 [file tx5c00562_si_001.zip › datasets_assay_IDs_random_and_scaffold_split_complete_results/scaffold_split_results/Permeability/6panel__permeability_bioassay__LightGBM__PAMPA.pdf]

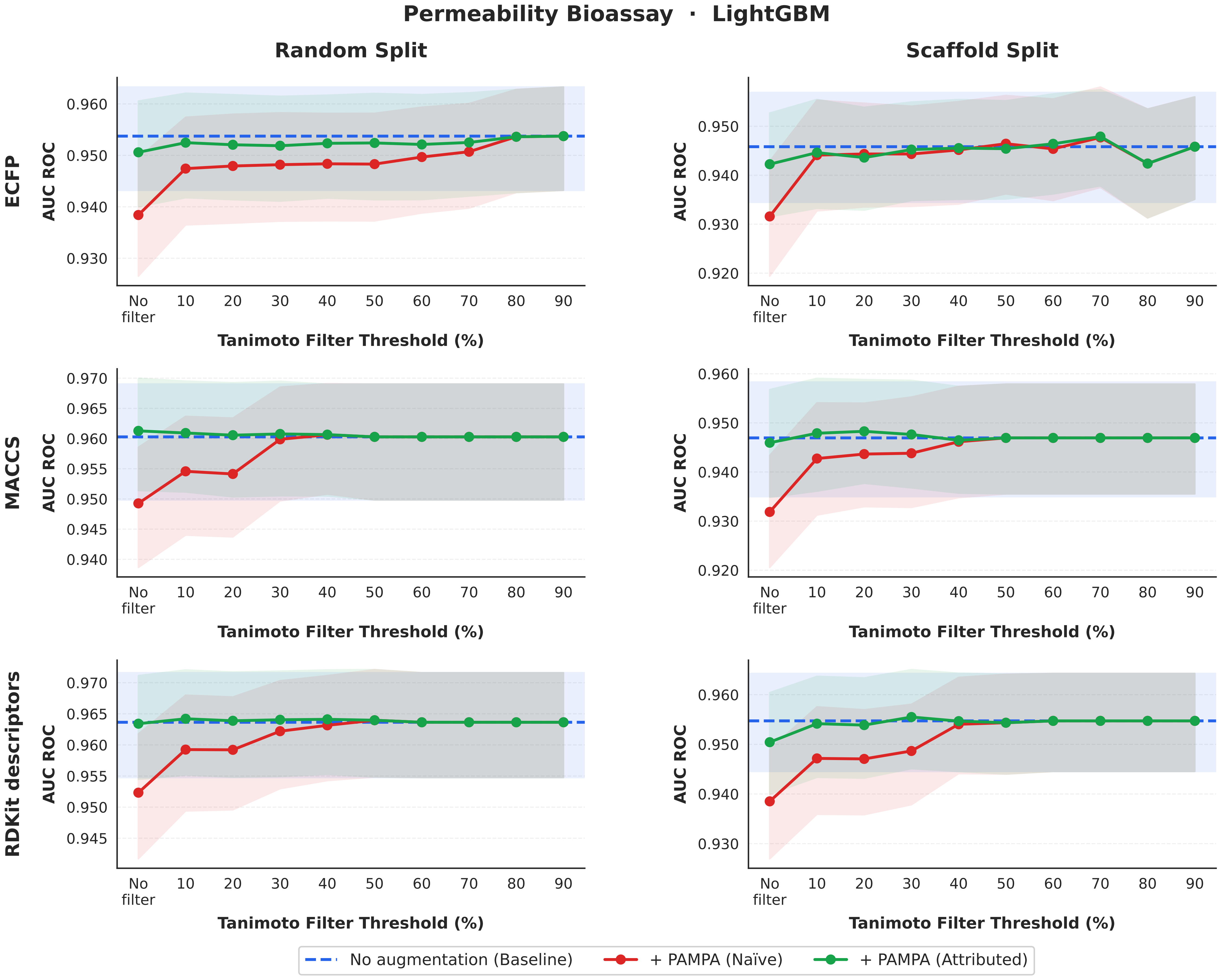

Permeability Bioassay · LightGBM
Random Split
Scaffold Split
ECFP
MACCS
RDKit descriptors
AUC ROC
Tanimoto Filter Threshold (%)
No filter
10
20
30
40
50
60
70
80
90
No augmentation (Baseline)
+ PAMPA (Naïve)
+ PAMPA (Attributed)
